# Minimal clinically important change of knee flexion in people with knee osteoarthritis after non-surgical interventions using a meta-analytical approach

**DOI:** 10.1186/s13643-023-02393-0

**Published:** 2024-02-01

**Authors:** M. Denika C. Silva, Andrew P. Woodward, Angela M. Fearon, Diana M. Perriman, Trevor J. Spencer, Jacqui M. Couldrick, Jennie M. Scarvell

**Affiliations:** 1 Faculty of Health, University of Canberra, Bruce, ACT 2617 Australia; 2 Trauma and Orthopaedic Research Unit, Canberra Hospital, Canberra, Australia; 3 Department of Physiotherapy, General Sir John Kotelawala Defence University, Werahera, Colombo, Sri Lanka; 4 Research Institute for Sport and Exercise, University of Canberra, Canberra, Australia; 5 College of Medicine and Health Sciences, Australian National University, Canberra, Australia

**Keywords:** Knee osteoarthritis, Knee flexion, Minimal clinically important change, Minimal clinically important difference, Non-surgical interventions, Errors-in-variables model, Bayesian meta-analysis, Hierarchical model, Meta-regression

## Abstract

**Background:**

Minimal clinically important change (MCIC) represents the minimum patient-perceived improvement in an outcome after treatment, in an individual or within a group over time. This study aimed to determine MCIC of knee flexion in people with knee OA after non-surgical interventions using a meta-analytical approach.

**Methods:**

Four databases (MEDLINE, Cochrane, Web of Science and CINAHL) were searched for studies of randomised clinical trials of non-surgical interventions with intervention duration of ≤ 3 months that reported change in (*Δ*) (mean change between baseline and immediately after the intervention) knee flexion with *Δ* pain or *Δ* function measured using tools that have established MCIC values. The risk of bias in the included studies was assessed using version 2 of the Cochrane risk-of-bias tool for randomised trials (RoB 2). Bayesian meta-analytic models were used to determine relationships between *Δ* flexion with *Δ* pain and *Δ* function after non-surgical interventions and MCIC of knee flexion.

**Results:**

Seventy-two studies (*k* = 72, *n* = 5174) were eligible. Meta-analyses included 140 intervention arms (*k* = 61, *n* = 4516) that reported *Δ* flexion with *Δ* pain using the visual analog scale (pain-VAS) and *Δ* function using the Western Ontario and McMaster Universities Osteoarthritis Index function subscale (function-WOMAC). Linear relationships between *Δ* pain at rest-VAS (0–100 mm) with *Δ* flexion were − 0.29 (− 0.44; − 0.15) (*β*: posterior median (*CrI*: credible interval)). Relationships between *Δ* pain during activity VAS and *Δ* flexion were − 0.29 (− 0.41, − 0.18), and *Δ* pain-general VAS and *Δ* flexion were − 0.33 (− 0.42, − 0.23). The relationship between *Δ* function-WOMAC (out of 100) and *Δ* flexion was − 0.15 (− 0.25, − 0.07). Increased *Δ* flexion was associated with decreased *Δ* pain-VAS and increased *Δ* function-WOMAC. The point estimates for MCIC of knee flexion ranged from 3.8 to 6.4°.

**Conclusions:**

The estimated knee flexion MCIC values from this study are the first to be reported using a novel meta-analytical method. The novel meta-analytical method may be useful to estimate MCIC for other measures where anchor questions are problematic.

**Systematic review registration:**

PROSPERO CRD42022323927.

**Supplementary Information:**

The online version contains supplementary material available at 10.1186/s13643-023-02393-0.

## Background

Minimal clinically important change (MCIC) represents the patient-perceived improvement in an outcome after treatment [1–4], in an individual or within a group over time [5–7]. Impaired knee flexion range of motion (flexion) is a characteristic clinical feature in people with knee OA [8] which impacts on function, mobility, quality of life and independence [9–11]. Moreover, restricted knee movement is one of the ten key signs recommended for diagnosing knee OA by the European Alliance of Associations for Rheumatology [12]. Knee flexion is a valid and convenient measure used in clinical and research settings. Therefore, estimates of MCIC of knee flexion are necessary to interpret treatment efficacy and calculate the sample size for research studies [13, 14].

The anchor method is the most frequently used method to calculate MCIC, where values are estimated using an external scale (termed ‘anchor’) [15, 16]. The global rating of change scale is commonly used as the ‘anchor’ in studies. For example, patients are asked a transition question after treatment: ‘overall, how are your hip or knee problems now, compared to before treatment?’ with response categories ‘much better’, ‘a little better’, ‘about the same’, ‘a little worse’ and ‘much worse’ [5, 6]. The MCIC is the average pre-post change score of the sub-group of patients who responded ‘a little better’. Some studies use multiple anchors to improve the validity of results [17, 18]; for example, using cut points for the Western Ontario and McMaster Universities Osteoarthritis Index (WOMAC), 36-Item Short-Form Health Survey and the Back Depression scale [19]. However, when selecting an anchor, the anchor should be relevant to the disease condition, clinicians should be familiar with it (clinical acceptance) and there should be a relationship between the anchor and the outcome measure being evaluated for MCIC [20, 21].

Knee flexion limitation is associated with joint pain and limited function [8, 9, 22]. Non-surgical interventions (for example physiotherapy, manual therapy and exercise) could improve knee flexion, relieve pain and improve function in people with knee OA [23–25]. Pain relief has been associated with knee flexion improvement in people with knee OA awaiting knee replacement [26]. Increased flexion has been related to improved function in people with knee OA after total knee replacement [27]. Therefore, change in (*Δ*, mean change between baseline and immediately after the intervention) knee flexion after treatment may be associated with *Δ* pain and *Δ* function.

The MCIC of knee flexion in people with knee OA has not been previously reported. MCIC estimates for pain and function in knee OA after non-surgical interventions have been established for several patient-reported outcome measures. These include the WOMAC function subscale (function-WOMAC) and the Visual Analogue Scale for pain (pain-VAS) [28, 29]. We hypothesised that the relationship between *Δ* flexion and *Δ* pain and/or *Δ* function measured by tools with known MCIC can be used to estimate the MCIC of knee flexion.

Therefore, this study aimed to use meta-analysis to infer the MCIC of knee flexion, using relationships between *Δ* knee flexion and *Δ* pain and/or *Δ* function measured by tools with known MCIC in people with knee OA after non-surgical interventions.

## Methods

We estimated the MCIC of knee flexion using a meta-analytical approach based on anchor method concepts. Data were obtained from published studies through a rapid systematic review, which was designed and reported according to the Preferred Reporting Items for Systematic reviews and Meta-Analyses (PRISMA) statement [30] and Cochrane rapid review guideline [31]. The protocol was registered on PROSPERO (registration number: CRD42022323927).

### Literature search

We searched in databases MEDLINE (EBSCO), Cochrane Central Register of Controlled Trials, Web of Science and Cumulative Index to Nursing and Statistics in practice Allied Health Literature (CINAHL) from inception up to September 2023. A comprehensive search strategy was developed (Additional file 1) to capture all relevant articles, and database-specific MESH terms were used. We limited our search to studies published in English, and grey literature was not searched. The records were exported to EndNote version X9.2 for reference management.

### Study screening

Covidence software (Covidence systematic review software, Veritas Health Innovation, Melbourne, Australia (www.covidence.org)) was used to manage the selection process. Records identified in the search were uploaded, and duplicates were removed. Two independent reviewers screened titles, abstracts and then full texts (D. S., T. S., J. C.). Disagreements were resolved by discussion with a third reviewer.

### Study selection criteria

We included randomised clinical trials of people with knee OA (diagnosed clinically or radiographically). Studies were excluded if the study included participants with knee OA together with participants with other disorders or OA in other joints that were not reported separately (Table 1). Studies included non-surgical interventions with a treatment duration of ≤ 3 months. We limited studies of treatment duration ≤ 3 months because estimates of MCIC are sensitive to treatment duration and follow-up time [13]. Studies were excluded if they reported on surgical interventions.

**Table 1:** Eligibility criteria of included studies

**Inclusion criteria**	**Exclusion criteria**
Randomised clinical trials including placebo-controlled trials, cluster-randomised trials and cross-over trials	Does not contain original data (e.g. systematic reviews, literature reviews and editorial commentaries)
Adults (aged 18 years or older) who have been diagnosed with knee osteoarthritis using any standard diagnostic criteria (clinically and/or radiographically)	Conference abstracts, research protocols and materials not published as a full peer-reviewed paper
Any nonsurgical intervention included (e.g. exercises, kinesio taping, massage therapy, herbal treatment and pharmacological)	Participants with knee osteoarthritis concurrently with participants with osteoarthritis in other joints unless data are reported separately
Treatment duration of 3 months or less	Participants with knee osteoarthritis concurrently with participants with other disorders such as rheumatoid arthritis, neck pain and back pain, unless data are reported separately
Estimate of change of knee flexion and patient-reported outcome measures^a^ immediately after the interventions	
Outcomes reported on pre- and post-intervention	

^a^The patient-reported outcome measures are selected that have known MCIC and MCID of outcome tools, namely *ICOAP*, intermittent and constant osteoarthritis pain

*KOOS* knee injury and osteoarthritis outcome score, *LEFS* Lower Extremity Functional Scale, *NPRS* Numeric Pain Rating Scale, *PROMIS* Patient-Reported Outcome Measurement Information System, *SF-36* 36-Item Short-Form Health Survey, *VAS* Visual Analog Scale, *WOMAC*, Western Ontario and McMaster Universities Arthritis Index

We included studies that reported *Δ* knee flexion as well as *Δ* pain or *Δ* function outcomes measured using tools with established MCICs [32]. The tools included intermittent and constant osteoarthritis pain, Knee injury and Osteoarthritis Outcome Score, Lower Extremity Functional Scale, Numeric Pain Rating Scale, Patient-Reported Outcome Measurement Information System, and 36-Item Short-Form Health Survey, VAS and WOMAC. We included studies that measured flexion using a universal goniometer, electrogoniometer or inclinometer in a static position as flexion assessed using the above tools is considered a valid and accessible outcome measure in knee OA [33–35]. Studies that assessed knee flexion during functional activities (for example gait, squatting, stair climbing) were excluded as they do not consider the full flexion range. Studies which reported *Δ* knee flexion as well as *Δ* pain or *Δ* function outcomes were included. However, if studies did not report mean change but reported mean values of pre-intervention (baseline) and post-interventions, they also were included. Studies that only reported effect sizes, median measures and only figures (did not report variance) were excluded.

### The risk-of-bias assessment

The risk of bias in the included studies was assessed using Version-2 of the Cochrane risk-of-bias tool for randomised trials (RoB 2) [36]. This instrument comprises five domains: bias arising from the randomisation process, bias due to deviations from intended interventions, bias due to missing outcome data, bias in the measurement of the outcome and bias in the selection of the reported result. Judgements for these domains and the overall risk of bias were estimated as ‘low, ‘high’ risk of bias or ‘unclear’. The review team considered the aim of the risk-of-bias assessment to assess the effects of assignment to intervention (the ‘intention-to-treat’ effect). When the assessment method of knee flexion is not reported in the primary article, the reference article or study protocol was checked to obtain that information. The risk of bias in all included studies was assessed by the principal investigator (D. S.), and a random sample of 35% of studies had a second review (T. S., J. C.) to improve the accuracy [37].

### Data extraction

Data extraction included the following: study design, sample size, characteristics of participants and details of the interventions and outcomes. We extracted mean change before and immediately after the intervention (*Δ*) and standard error (SE) of flexion, pain and function outcomes. If studies did not report mean change (inferential statistics), we extracted mean values of pre-intervention (baseline) and post-intervention (descriptive statistics). Knee flexion change was extracted for the index knee (the most affected knee). If the index knee could not be identified (for example in bilateral knee OA), both right and left knee flexion data were extracted. The principal investigator (D. S.) performed data extraction.

### Data analysis

First, we standardised *Δ* knee flexion of the index knee, *Δ* pain and *Δ* function. Where studies reported flexion data in both knees, average flexion was calculated. If a study did not report *Δ* flexion, it was calculated by subtracting pre-mean and post-mean according to Chapter 6.5.2.8 in the Cochrane handbook [38]. While this is likely to overstate the data (assuming those values are independent of each other as pre-mean is not predictive of post-mean), this is the only possible way to calculate the mean change using the given data of most studies. If the SE of mean change was not reported, it was calculated from standard deviation (SD) or confidence intervals (CI) using standard methods [38].

If studies reported pain-VAS in different scales, for example 0 to 10 mm or 0 to 100 mm, all the pain-VAS scores were standardised into a 0 to 100 mm scale where 0 = no pain at all and 100 = worst pain. All function-WOMAC scores were standardised to a 0 to 100 scale (0 = best function and 100 = worst function) where reported as 0 to 17 or 0 to 65.

Second, meta-analyses were performed to estimate the relationships between *Δ* flexion and *Δ* pain and/or *Δ* function. When a study had two or more interventions, each intervention arm was included as a separate cohort because each cohort was independent. However, study arms without interventions, for example waiting for treatment, were excluded. When studies reported both active and passive flexion, we included active flexion data only to avoid overestimating the same participants [38]. For this study, pain was examined in three categories: rest, during activity and general. Where pain was reported as ‘pain’, ‘pain intensity’ or ‘pain level’ without qualifiers, they were categorised to pain-general.

Only non-surgical intervention studies were included, but they comprised pharmacological, non-pharmacological or mixed interventions (type of interventions). Knee flexion can be measured in supine or prone (position) and actively or passively (mode). However, some studies did not provide this level in detail (missing data). Therefore, we examined the sensitivity of the data to these factors using data visualisation techniques and determined whether all data could be pooled or whether it should be analysed separately. Where there was no apparent effect due to these factors, we pooled knee flexion data regardless of the type of intervention, position or mode. In this way, the papers with missing data with regard to position or mode could be included in the pooled analysis.

To estimate relationships between *Δ* flexion and *Δ* pain and/or *Δ* function, meta-analytical models were developed using ‘R’ software [39] using the ‘brms’ package [40]. Separate analyses were conducted for each relationship, e.g. *Δ* flexion and *Δ* pain at rest and *Δ* flexion and *Δ* pain during activity. The Bayesian hierarchical models were used to determine relationships between variables: *Δ* flexion (Y, response) with* Δ* pain or *Δ* function (X, predictor). Errors-in-variables models were developed because both the predictor and the response variables have measurement errors [41]. We considered flexion as the response variable and pain or function as predictors for the convenience of implementing MCIC of knee flexion.

### Model in general form

$$\Delta flexion\sim {N({\beta }_{0},{\tau }_{study})+(\beta }_{pain}\cdot \Delta pain)+N(0,{\tau }_{arm}),{\Delta pain}_{j}\sim N({{\mu }_{pain}}_{j},{{\sigma }_{pain}}_{j})$$

In this model, $\Delta flexion$ and $\Delta pain$ have uncertainty components, and ${\beta }_{0}$ is the population intercept. ${\tau }_{study}$ represents the heterogeneity between-study variation, and ${\tau }_{arm}$ represents the heterogeneity between intervention arms.

${\Delta pain}_{j}\sim N({{\mu }_{pain}}_{j},{{\sigma }_{pain}}_{j})$: Latent variable representing the true value of the* j* (any given study) is being estimated based on the mean (*μ)* and standard error (*σ*). The above model is expressed as the following code.

#### Example model

$$(\mathrm{bf}\ (\mathrm{flexion}.\mathrm{change}\ |\mathrm{se}\ (\mathrm{SE}\_\mathrm{flexion}.\mathrm{change})\sim 1 +\mathrm{ me}\ (\mathrm{function}.\mathrm{change},\mathrm{ SE}\_\mathrm{function}.\mathrm{change}) + (1|\mathrm{Study}) + (1|\mathrm{Study}:\mathrm{Groups}))$$

In this model syntax (Bayesian regression model), ‘(1|Study) + (1|Study:Groups)’ indicates intervention arms (included hierarchical or ‘random’ effects), which are correlated across responses (univariate-normal distribution of intercepts).

Priors were intended to be weakly informative. The coefficient prior *β* was $\mathrm{N}(\mathrm{0,1})$; on the response scale, this suggests a maximal effect of a two-unit change in flexion for a one-unit change in the predictor. The maximum physiologic flexion range of the knee joint is about 135°. We presumed that the maximal effect of 60° in flexion 60/100 change in the pain and 60/100 function could be possible. Posterior mean and credible intervals (*CrI*) were implemented and visualised for all analyses using the packages ‘ggdist’ [42] and ‘ggplot2’ [43]. Heterogeneity had a normal prior distribution with mean and standard deviation ($\tau$, standard deviation of the between-study variability and between intervention arms). $\tau$ had a half-Cauchy hyperprior, HC (0, 1), intended to be weakly informative [44]. Finally, the goodness of fit of the model was assessed by a posterior predictor check. Relationships are presented as the slope (*β*), intercepts and heterogeneity between studies and intervention arms with 90% *CrI*.

Third, the MCIC of flexion was estimated if only the uncertainty of the above relationships was sufficiently low. To estimate the MCIC of knee flexion, we post-processed the above models with the established MCIC estimates for pain and function. For example, MCIC of pain-VAS = − 19.9 (− 21.6 to − 17.9) in a 0–100-mm scale [45], MCIC of function-WOMAC (out of 100) = − 9.1 (− 10.5 to − 7.5) [45], − 17.13 (− 20.07 to 14.19) [29] and − 17.02 (− 20.15 to − 13.9) [29].

### MCIC estimates for knee flexion using only reported-supine knee flexion data

However, as the supine position is frequently considered the appropriate knee flexion measurement position [46], a separate analysis was performed, including only reported-supine knee flexion data. First, relationships between Δ supine-active flexion with Δ pain and Δ function were established, and MCIC estimates of supine-active flexion were estimated as above. Second, relationships of pooled-supine flexion (mode = supine and position = active, passive or position not reported) with Δ pain and Δ function were also established. Then, MCIC estimates of pooled-supine flexion were estimated as above.

## Results

### Study selection

**Fig. 1:**
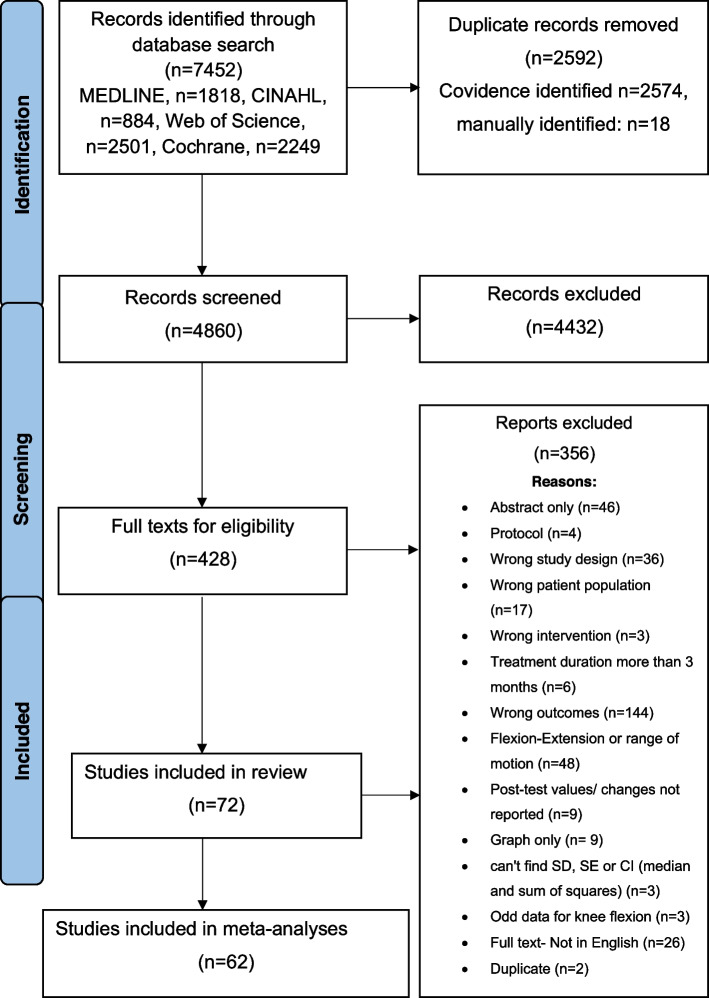
PRISMA flow diagram

The search yielded 7452 records; 4860 records were screened after removing duplicates. Title and abstract screening yielded 428 records. After screening full texts, 72 studies (*k* = 72, sample *n* = 5174) (Fig. 1) were identified that reported *Δ* flexion with *Δ* pain or *Δ* function. A list of references for included studies is available in Additional file 2.

There was a sufficient number of studies [37] to determine the relationships between *Δ* flexion with Δ pain using VAS (pain-VAS) and *Δ* function using WOMAC function subscale (function-WOMAC) only. Therefore, meta-analyses included 61 studies with 140 study arms (*k* = 61, *n* = 4516) that reported *Δ* flexion and *Δ* pain-VAS or *Δ* function-WOMAC. Eleven studies (Table 2) reported *Δ* pain or *Δ* function using other tools, for example Numeric Pain Rating Scale and Knee Injury and Osteoarthritis Outcome Score.

**Table 2:** Study characteristics

**Study **	**Knee OA diagnosis, KL**	**Treatment arms** **(Treatment category)**	**Duration** **(Assessment time point)**	**Participants** **Mean Age (SD)** **Mean BMI (SD)**	**Outcome measures**
**Abolhasani et al., 2019** [47]	ACR KL: II to III	G1: Kinesiotape (Non-pharmacological)	3 days (3 days)	*n *= 14 57.5 (6.7); 28.4 (5.3)	Knee flexion- Standard goniometry Pain (VAS)- Pain intensity
		G2: Sham-kinesiotape (Non-pharmacological)		*n *= 13 57.2 (8.8); 27.6(4.8)	
**Alfredo et al., 2012** [48]	ACR II to IV	G1: Laser therapy with exercises (Non-pharmacological)	3 weeks (3 weeks)	*n *= 20 61.2 (7.5); 30.2 (4.1)	Knee flexion- Universal goniometer Pain (VAS)-Pain WOMAC- Pain, Function, Stiffness, Total
		G2: Placebo laser therapy with exercises (Non-pharmacological)		*n *= 22 62.3 (6.9); 29.2 (5)	
**Alfredo et al., 2020** [49]	ACR II to IV	G1: Continuous ultrasound therapy at the 1st month + exercises at the 2nd month (Non-pharmacological)	8 weeks (8 weeks)	*n *= 20 64.4 (6.2); 31 (3.4)	Knee flexion- Universal goniometer Pain (VAS)- ADL, rest WOMAC- Pain, Function, Stiffness, Total
		G2: Pulsed ultrasound therapy at the 1st month+ exercises at the 2nd month (Non-pharmacological)		*n *= 20 63.9 (4.5); 27.8 (3.7)	
		G3: Continuous ultrasound therapy at the 1st month + Continuous ultrasound therapy with exercises (Non-pharmacological)		*n* = 20 65.8 (6); 26.5 (6.6)	
		G4: Pulsed ultrasound therapy at the 1st month + Pulsed ultrasound therapy with exercises (Non-pharmacological)		*n* = 20 66.7 (6.6); 27.9 (4.2)	
		G5: Exercises (Non-pharmacological)		*n *= 20 62.7 (8.5); 31.1 (3.2)	
**Alkhawajah & Alshami, 2019** [25]	ACR II to IV	G1: Mobilisation with movement (Non-pharmacological)	1 session (immediately)	*n *= 20 56.5 (7.6); 32.6 (7.8)	Knee flexion- Goniometer Pain (VAS)- Current pain intensity WOMAC- Function, Stiffness, Total
		G2: Sham Mobilisation with movement, active passive movement 10 times, 3 sets (Non-pharmacological)		*n* = 20 56.6 (8.5); 33.3 (6.1)	
**Altınbilek et al., 2018** [50]	ACR II to III	G1: Osteopathic manipulative treatment (Non-pharmacological)	2 Weeks (2 Weeks)	*n* = 44 53.9 (8.2) 32.3 (5.2)	Knee flexion- Standard goniometer WOMAC- Pain, Function, Stiffness
		G2: Exercise group (Non-pharmacological)		*n* = 41 55.6 (8.8); 30.9 (5.9)	
**Alpay and Sahin 2022** [51]	Radiographic II to III	G1: Basic body awareness therapy+ Home exercise program (Non-pharmacological)	6 Weeks (6 Weeks)	*n *= 20 53.7 (5.7), 31.8 (4.9)	Knee flexion- Standard goniometer Pain (VAS)-Pain intensity WOMAC-total
		G2: Home exercise program (Non-pharmacological)		*n *= 20 56.3 (6.3), 32.1 (5.9)	
**Arslan and Kul, 2022** [52]	ACR II to III	G1: Shockwave treatment (Non-pharmacological)	3 Weeks (10 days)	*n *= 26 57.4 (8.3); 34.1 (5.0)	Knee flexion- Standard goniometer Pain (VAS)- Pain WOMAC- Pain, Function, Stiffness, Total
		G2: Conventional physical therapy (Non-pharmacological)		*n *= 26 57.4 (8.3); 34.1 (5.0)	
**Ashraff et al., 2022**^**a**^ [53]	Radiographic (0 to IV)	G1: Low level laser therapy combined with conventional exercises	3 weeks (3 weeks)	*n *= 22 52.8 (3.0); 29.3 (1.4)	Knee flexion- Goniometer WOMAC-total Pain (NPRS)- Pain
		G2: Conventional exercises only.		*n *= 22 53.0 (3.3); 29.5 (1.6)	
**Askari et al., 2019** [54]	ACR I to III	G1: Sesame oil massage therapy (Non-pharmacological)	4 weeks (4 weeks)	*n *= 47 57.5 (12.5); 28.7 (3.8)	Knee flexion- Standard goniometer Pain (VAS)- joint pain WOMAC- Pain, Function, Stiffness, Total
		G2: Diclofenac sodium gel therapy (Pharmacological)		*n *= 47 57.5 (9.1); 27.8 (4.5)	
**Assar et al., 2020** [55]	ACR II to IV	G1: Total resistance exercises (Non-pharmacological)	8 weeks (8 weeks)	*n *= 12 55.9 (8.6); 29.8 (7.2)	Knee flexion- Bubble inclinometer device Pain (VAS)- Pain at daily activities WOMAC- Stiffness
		G2: Aquatic exercises (Non-pharmacological)		*n* = 12 57.5 (6.9); 28.5 (3.7)	
		G3: Control group: rheumatologic advice only (No treatment)		*n *= 12 63.8 (7.5); 23.1 (11.6)	
**Aydoğdu et al., 2017** [56]	ACR II to III	G1: Kinesiotape + UST + TENS + Electrotherapy + Cold pack therapy (Non-pharmacological)	3 weeks (3 weeks)	*n *= 28 52.5 (9.7); 31.2 (5.1)	Knee flexion- Universal goniometer Pain (VAS)- Pain level KOOS- Symptoms, Pain, ADL, Sports and recreation, QOL
		G2: Control group: UST + TENS + Electrotherapy + Cold pack therapy (Non-pharmacological)		*n *= 26, 51.2 (8.9); 31.5 (5.7)	
**Babaskin et al., 2019** [57]	ACR I to II	G1: Phyto complex electrophoresis + drug therapy (Mixed)	10 days (10 days)	*n *= 36 NR, NR	Knee flexion- Goniometer WOMAC- Pain, Function, Stiffness
		G2: Amplipulse therapy + drug therapy (Mixed)		*n *= 36 NR, NR	
		G3: Drug therapy: Slow Acting Drugs for Osteoarthritis (Pharmacological)		*n *= 36 NR, NR	
**Benedetti et al., 2017** [58]	ACR NR	G1: Local Muscle Vibration therapy (non-pharmacological)	2 weeks (2 weeks)	*n *= 15 61.8 (5.8); 26.1 (2.9)	Knee flexion- Instrument- NR Pain (VAS)-Pain WOMAC-Total
		G2: Neuromuscular Electrical Stimulation (Non-pharmacological)		*n *= 15 55.7 (9.1); 26 (2.8)	
**Bhore and Shinde**^**a**^**, 2023** [59]	Radiographic I to II	G1: Multi‐Component Exercise Program (Non-pharmacological)	6 weeks (6 weeks)	*n* = 60 NR; NR	Knee flexion- Goniometer WOMAC- Total
		G2: Conventional Group (electrotherapy modalities like the hot moist pack, interferential therapy, ultrasonic therapy, active ROM exercises and progressive resistance exercises). (Non-pharmacological)		*n *= 60 NR; NR	
**Peréz Busquier et al., 1997** [60]	WHO NR	G1: Aceclofenac tablets (Pharmacological)	2 months (2 months)	*n* = 123 59.3 (8.9); nr	Knee flexion- Goniometer Pain (VAS)-Pain
		G2: Piroxicam tablets (Pharmacological)		*n* = 117 59.7 (7.4); nr	
**Coleman et al., 2012** [61]	Clinical NR	G1: Knee self-management program (Non-pharmacological)	6 weeks (8 weeks)	*n* = 71 65 (7.9); nr	Knee flexion- Goniometer Pain (VAS)-Pain WOMAC- Pain, Function, Stiffness, Total SF36-Physical function, Role physical, Body pain, General health, Vitality, Social function, Role emotional, Mental health Pain (NPRS)- Pain
		G2: Control group: waiting period (No treatment)		*n* = 75 65 (8.7); nr	
**Costa et al., 2020** [62]	Radiographic I to III	G1: UCII (Undenatured Oral type II Collagen) and physiotherapy (Mixed)	1 month (1 month)	*n *= 20 55.5 (8.8); 30.2 (4.9)	Knee flexion- Goniometer Pain (VAS)- Pain WOMAC- Total
		G2: Placebo UCII and physiotherapy (Mixed)		*n* = 20 57.4 (11.4); 30.3 (5.7)	
		G3: Physiotherapy (Non-pharmacological)		*n *= 20 59.6 (8.2); 30.4 (5.8)	
**Deniz et al., 2009** [63]	ACR II to IV	G1: Continuous diclofenac gel phonophoresis (Mixed)	10 days (10 days)	*n* = 20 56.4 (6.5); 31.2 (4.5)	Knee flexion- Instrument- NR Pain (VAS)- Pain at rest, in activity WOMAC- Pain, Function, Stiffness
		G2: Pulsed diclofenac gel phonophoresis (Mixed)		*n* = 20 57.1 (6.6); 30.1 (4.3)	
		G3: Diclofenac gel sham phonophoresis (Mixed)		*n* = 20 54.5 (6.8); 30.6 (4.1)	
		G4: Acoustic gel sham phonophoresis (Non-pharmacological)		*n* = 20 56.3 (6.7); 29.1 (3.8)	
**Dogan et al; 2022** [64]	ACR >II	G1: Kinesio taping (Non-pharmacological)	7 weeks (7 weeks)	*n* = 27 56.9 (6.9); 32.8 (5.8)	Knee flexion- Instrument- NR Pain (VAS)- Pain at rest, in activity
		G2: Sham kinesio taping (Non-pharmacological)		*n* = 30 55.7 (6.9); 30.8 (5.4)	
**Donec & Kubilius, 2020**^**a**^ [65]	Radiographic I to III	G1: Kinesiotaping (Non-pharmacological)	4 weeks (4 weeks)	*n* = 81 (123 knees 68.7 (9.9); 30.5 (5.3)	Knee flexion- Goniometer KOOS- Symptoms, Pain, ADL, Sports and recreation, QOL
		G2: Non-specific kinesiotaping (Non-pharmacological)		*n *= 76 (114 knees) 70.6 (8.3); 30.7 (5.2)	
**Draper et al., 2018** [66]	Radiographic I to II	G1: Low intensity long duration ultrasound therapy (Non-pharmacological)	6 weeks (6 weeks)	*n *= 51 53.6 (8.9); 34.9 (8.9)	Knee flexion- Inclinometer WOMAC- Pain, Function, Stiffness, Total Pain (NPRS)- Pain
		G2: Placebo ultrasound therapy (Non-pharmacological)		*n *= 31 51 (9); 34.5 (8.3)	
**Dwyer et al., 2015** [67]	ACR 0-III	G1: Mobilisation with movement (Non-pharmacological)	4 weeks (4 weeks)	*n *= 26 63.5 (10.9); 28.6 (5.2)	Knee flexion- Digital Inclinometer WOMAC- Pain, Function, Stiffness, Total
		G2: Rehabilitation program (Non-pharmacological)		*n *= 26 60.9 (10.3); 30.8 (6.4)	
		G3: Mobilisation with movement plus rehabilitation (Non-pharmacological)		*n *= 26 62.2 (11.8); 30.6 (7.6)	
**Eftekharsadat et al., 2020** [68]	ACR II to III	G1: Shock wave therapy (Non-pharmacological)	3 weeks (3 weeks)	*n *= 25 58 (6); NR	Knee flexion- Goniometer Pain (VAS)- Pain intensity at rest WOMAC- Pain, Function, Stiffness, Total
		G2: Physiotherapy (Non-pharmacological)		*n *= 25 55.8 (6.1); NR	
		G3: Exercise therapy (Non-pharmacological)		*n *= 25 58.2 (7.2); NR	
**Elgendy et al., 2020** [69]	Clinical and raiographic II to III	G1: Shock wave therapy (Non-pharmacological)	4 weeks (4 weeks)	*n *= 15 48.7 (8.6); 31.3 (2.3)	Knee flexion- Laser Goniometer Pain (VAS)- Pain intensity WOMAC- Total
		G2: Platelet rich plasma injection (Pharmacological)		*n *= 15 49.2 (9.2); 31.5 (2.0)	
		G3: Conventional physiotherapy (Stretching and strengthening+UST+hot pack) (Non-pharmacological)		*n *= 15 55.1 (6.7); 30.8 (2.5)	
**ElGendy et al., 2022** [70]	Radiographic II to III	G1: Rectus femoris stretching exercises together with stretching exercises of the calf, hamstring and iliotibial band, strength exercises for the quadriceps, gluteus medius, gluteus maximus and calf muscles (Non-pharmacological)	4 weeks (4 weeks)	*n *= 30 53.6 (6.0); 32.1 (0.8)	Knee flexion- Universal goniometer Pain (VAS)- Pain intensity WOMAC- Total
		G2: Exercises mentioned for G1 except rectus femoris stretching. (Non-pharmacological)		*n *= 30 53.1 (5.9); 32.1 (0.7)	
**Fakhari et al., 2021** [71]	Radiographic II to III	G1: Ozone intraarticular injection (Non-pharmacological)	4 weeks (4 weeks)	*n *= 30, 57.7 (9.4); NR	Knee flexion- Goniometer Pain (VAS)- Pain WOMAC- Total
		G2: Low level lazer therapy (Non-pharmacological)		*n *= 30 53.3 (9.4); NR	
**Sousa Filho et al., 2017** [72]	Clinically NR	G1: Ultrasound therapy (Non-pharmacological)	5 weeks (5 weeks)	*n *= 30, 61.8 (12.5); 29 (2.4)	Knee flexion- Goniometer Pain (VAS)- Pain intensity
		G2: Ultrasound and capaiba oil massage (Mixed)		*n* = 30 61.1 (8.2); 29.4 (7.2)	
		G3: Capaiba oil massage (Pharmacological)		*n *= 30 61.4 (9.9); 34.5 (6.2)	
**Fish et al., 2008** [73]	Clinically and radiographic NR	G1: Capasaicin massage (Pharmacological)	3 weeks (3 weeks)	*n* = 20 62 (SD); NR	Knee flexion- Goniometer Pain (NPRS)- Pain WOMAC-Total
		G2: Maitland mobilisation (Non-pharmacological)		*n *= 20 60 (SD); NR	
		G3: Maitland mobilisation with capasaicin massage (Mixed)		*n* = 20 62 (SD); NR	
**Forogh et al., 2016** [74]	ACR II to III	G1: Intraarticular platelet rich plasma (Pharmacological)	1 session (2 months)	*n* = 24 59.1 (7.0); 28.9 (2.8)	Knee flexion- Goniometer Pain (VAS)- Pain intensity KOOS- Pain, Symptoms, ADL, QOL, Sports
		G2: Intraarticular corticosteroid (Pharmacological)		*n* = 24 61.1 (6.7); 29.2 (3.4)	
**Fung, 2021** [75]	Arden and Nevitt NR	G1: Passive mobilisation with movement with a machine (PANDA) (Non-pharmacological)	2 weeks (2 weeks)	*n *= 34 75.1 (8); 22 (2)	Knee flexion- Electronic Goniometer Pain (VAS)- Pain intensity WOMAC- Pain, Function, Stiffness, Total
		G2: Control group: Sham PANDA and exercise (Non-pharmacological)		*n* = 34 76.1 (7.5); 22.5 (2.2)	
**Gungen et al., 2012** [76]	ACR III to IV	G1: Hot pack therapy (Non-pharmacological)	2 weeks (2 weeks)	*n *= 21 61.9 (6.7); 27.6 (2.4)	Knee flexion- Goniometer Pain (VAS)- Pain at rest, at night, activity WOMAC- Pain, Function, Stiffness
		G2: Mud pack therapy (Non-pharmacological)		*n *= 23 65 (7.1); 28 (2.8)	
**Gur et al., 2003** [77]	ACR II to IV	G1: Acute laser therapy: 5 minutes/3J+exercises (Non-pharmacological)	14 weeks (12 weeks)	*n *= 30 58.6 (5.9); 31.2 (3.8)	Flexion- Goniometer Pain (VAS)- Pain at rest, at movement, at flexion WOMAC- Total
		G2: Acute laser therapy: 3 minutes/2J+exercises (Non-pharmacological)		*n* = 30 59.8 (8.0); 28.5 (3.0)	
		G3: Placebo laser therapy+exercises (Non-pharmacological)		*n* = 30 60.5 (6.9); 30.3 (3.1)	
**Gurudut & Jaiswal, 2022**^**a**^ [78]	Radiographic III to IV	G1: Progressive muscle relaxation (Non-pharmacological)	2 weeks (2 weeks)	*n* = 5 55.8 (6.3); 33.3 (4.9)	Flexion- Universal goniometer WOMAC- Total
		G2: Yoga graded motor imagery (Non-pharmacological)		*n* = 6 45.8 (8.4); 37.7 (6.3)	
**Hewlings et al., 2019** [79]	ACR NR	G1: Hydroselate dietary supplement (Pharmacological)	12 weeks (4 weeks)	*n* = 44 NR; NR	Standard goniometer (JAMA) WOMAC- Pain, Function, Stiffness
		G2: Placebo group: same excipients but without the active study product (Pharmacological)		*n* = 44 NR; NR	
**Ho et al., 2021** [80]	radiographic IV	G1: Thermal gun (Non-pharmacological)	4 weeks (4 weeks)	*n* = 38 68 (6.2); NR	Flexion- Goniometer Pain (VAS)- Pain WOMAC- Pain, Function, Stiffness, Total
		G2: Heat pack treatment (Non-pharmacological)		*n *= 38 66.6 (7.4); NR	
**Kaya Mutlu et al., 2017** [81]	ACR II to IV	G1: Kinesiotaping (Non-pharmacological)	3 sessions (3sessions)	*n* = 20 54.3 (6); 30.7 (3.8)	Flexion- Digital goniometer Pain (VAS)- Pain intensity-at rest, activity, night WOMAC- Total
		G2: Placebo kinesiotaping (Non-pharmacological)		*n *= 19 57.1 (6.3); 31.3 (6.2)	
**Kaya Mutlu et al., 2018** [24]	ACR II to III	G1: Mobilisation with movement (Non-pharmacological)	4 weeks (4 weeks)	*n* = 21 54.2 (7.3); 30.8 (5.0)	Flexion- Digital goniometer Pain (VAS)- Pain intensity-at rest, activity, night WOMAC- Pain, Function, Stiffness, Total
		G2: Passive joint mobilisation (Non-pharmacological)		*n* = 21 56.3 (6.6); 30.7 (4.3)	
		G3: Electrotherapy (Non-pharmacological)		*n* = 22 57.8 6.2); 32.6 (5.7)	
**Khademi-Kalantari et al., 2014** [82]	Clinical and radiographic III to IV	G1: Physiotherapy + knee distraction (Non-pharmacological)	2 weeks (2 weeks)	*n* = 20 61.2 (8.6); 32.3 (5.6)	Flexion- Goniometer Pain (VAS)- Pain KOOS- Pain, Symptoms, Activity, Sports and recreation, QOL
		G2: Physiotherapy (Non-pharmacological)		*n* = 20 61.3 (6.8); 30.8 (6.1)	
**Kus et al., 2023** [83]	ACR II to III	G1: Sensory-motor training (Non-pharmacological)	8 weeks (8 weeks)	*n* = 24 58.5 (7.1); 30.5 (3.9)	Flexion- Digital goniometer Pain (VAS)- Pain-rest, activity, night WOMAC- Total
		G2: Resistance training (Non-pharmacological)		*n* = 24 60.0 (6.1); 29.6 (3.8)	
**Lizis et al., 2017** [84]	ACR NR	G1: Extracorporeal shockwave therapy (Non-pharmacological)	5 weeks (5 weeks)	*n* = 20 63.5 (8); 24.9 (1.9)	Flexion- Goniometer WOMAC- Pain, Function, Stiffness, Total
		G2: Kinesiotherapy (Non-pharmacological)		*n* = 20 65.0 (8.4); 24.1 (1.5)	
**Lizis et al., 2019** [85]	ACR NR	G1: Manual therapy with cryotherapy (Non-pharmacological)	5 weeks (5 weeks)	*n* = 64 62.3 (9.7); 24.2 (1.8)	Flexion- Goniometer Pain (VAS)- Current level- pain WOMAC- Pain, Function, Stiffness
		G2: Kinesiotherapy with cryotherapy (Non-pharmacological)		*n* = 64 62 (11.4); 23.6 (1.7)	
**Lun et al., 2015** [86]	Clinical and radiographic NR	G1: Hip strengthening exercises (Non-pharmacological)	12 weeks (12 weeks)	*n* = 37 63.4 (9.6); 29.3 (4.2)	Flexion- Goniometer WOMAC- Pain, Function, Stiffness KOOS- Pain, Symptoms, ADL, QOL, Sports recreation
		G2: Leg strengthening exercises (Non-pharmacological)		*n* = 34 61.38 (7.7); 30.8 (6.3)	
**Ma et al., 2023** [87]	Radiographic (II to IV)	G1: Dry needling latent and active myofascial trigger point with the stretching group (Non-pharmacological)	6 weeks (6 weeks)	*n* = 42 74.6 (6.4); 26.1 (3.4)	Flexion- Goniometer WOMAC- Pain, Function, Stiffness Pain (NPRS)-Pain severity
		G2: Oral diclofenac with the stretching group (Mixed)		*n* = 35 75.4 (5.8); 24.5 (3.3)	
**Mendes et al., 2019** [88]	ACR II to III	G1: Botulinum toxin intraarticular injection (Pharmacological)	1 session (4 weeks)	*n* = 35 62.5 (6.8); 30.4 (4.8)	Flexion- Instrument- NR VAS(Pain)- Rest, activity WOMAC- Pain, Function, Stiffness, Total
		G2: Triaminolone hexacetonide intraarticular injection (Pharmacological)		*n* = 35 65.5 (6.9); 32.7 (5.5)	
		G3: Saline solution intraarticuar injection (Pharmacological)		*n* = 35 64.6 (6.7); 30.5 (5.3)	
**Moezy et al., 2021** [89]	ACR II to III	G1: Exercise to activate vastus medialis (Non-pharmacological)	4 weeks (4 weeks)	*n* = 33 55.5 (4.3); 26.8 (2.3)	Flexion- Goniometer VAS(Pain)- Pain severity
		G2: Conventional exercise (Strengthening, stretching non-specific) (Non-pharmacological)		*n* = 32 56.6 (6.3); 25.8 (2.2)	
**Mohamed and Alatawi, 2022** [90]	Clinical and radiographic NR	G1: Kinesio taping plus conventional physical therapy (Non-pharmacological**)**	6 weeks (6 weeks)	*n* = 20 60.6 (9.4); 26.7 (2.9)	Flexion- Goniometer Pain (NPRS)- Pain intensity WOMAC- Pain, Function, Stiffness
		G2: Conventional physical therapy (Non-pharmacological**)**		*n* = 20 63.4 (8.0); 27.7 (2.4)	
**Nam et al., 2014**^**a**^ [91]	Clinical III to IV	G1: Exercise on unstable surface (Non-pharmacological)	6 weeks (6 weeks)	*n* = 15 64.9 (6.8); NR	Flexion- Standard goniometer WOMAC- Total
		G2: Exercise on stable surface (Non-pharmacological)		*n* = 15 63.7 (5.6); NR	
**Nazari et al., 2019** [92]	ACR II to III	G1: High intensity laser therapy combination with exercises (Non-pharmacological)	4 weeks (4 weeks)	*n* = 30 61.5 (3.9); 27.7 (1.4)	Flexion- Instrument- NR Pain (VAS)- Pain intensity WOMAC- Pain, Function, Stiffness, Total
		G2: Conventional physiotherapy (TENS+UST) in combination with exercise (Non-pharmacological)		*n* = 30 62.4 (3.1); 27.2 (1.6)	
		G3: Exercise alone (Non-pharmacological)		*n* = 30 62.2 (3.9); 27.5 (1.8)	
**Nidup et al., 2020**^**a**^ [93]	Clinical I to II	G1: Rsta-byugs application (Pharmacological)	1 week (1 week)	*n* = 31 62.8 (7.2); 62.6 (8.4)	Flexion- Goniometer KOOS- Pain, Symptoms, ADL, QOL, Sports recreation
		G2: Diclofenac gel application (Pharmacological)		*n* = 31 25.1 (3.9); 25.7 (4.2)	
**Oktayoglu et al., 2014** [94]	ACR II to IV	G1: Phonophoresis (diclofenac sodium) (Mixed)	10 sessions (15 day)	*n* = 20 54.6 (8.7); 29.7 (4.2)	Flexion- Goniometer Pain (VAS)- Walking, rest, flexion WOMAC- Pain, Function, Stiffness, Total
		G2: Conventional Ultrasound (Non-pharmacological)		*n* = 20 55.1 (10.1); 30.2 (3.3)	
**Parfitt & Parfitt, 2006** [95]	Clinical NR	G1: Intraarticular corticosteroid injection+ home exercise (Mixed)	8 weeks (8 weeks)	*n* = 8 76.13 (7.74); NR	Flexion- Goniometer Pain (VAS)- Pain at rest, after walking, at night, worst pain WOMAC- Pain, Function, Stiffness, Total
		G2: Intraarticular corticosteroid injection (Pharmacological)		*n* = 5 67.2 (6.01); NR	
**Parmigiani et al., 2010**^**a**^ [96]	ACR II to III	G1: Joint lavage followed by intra-articular injection with triamcinolone hexacetonide (Pharmacological)	1 session (1 session)	*n* = 30 66.2 (9.07); 31.1 (5.1)	Flexion- Goniometer WOMAC- Pain
		G2: Intra-articular injection with triamcinolone hexacetonide (Pharmacological)		*n* = 30 61.2 (7.3); 32.4 (6)	
**Petrella & Petrella, 2006** [97]	Radiographic I to III	G1: Hyaluronic acid intra articular injection (Pharmacological)	3 weeks (6 weeks)	*n* = 51 63.9 (9.3); NR	Flexion- Goniometer Pain (VAS)- Pain-walking, stepping WOMAC- Pain, Function, Stiffness SF36- Physical function, Role physical, Body pain, General health, Vitality, Social function, Role emotional, Mental health
		G2: Placebo/ saline injection (Pharmacological)		*n* = 54 62.4 (10.3); NR	
**Pinkaew et al., 2019** [98]	ACR NR	G1: Ultrasound therapy (Non-pharmacological)	10 days (10 days)	*n *= 20 64.3 (9.7); 22.6 (8.6)	Flexion- Plastic goniometer Pain (VAS)- Pain WOMAC- Severity of pain SF36- Total
		G2: Phonophoresis of phyllanthus amarus (Mixed)		*n *= 20 65.2 (8.3); 23.6 (5.2)	
**Prasad et al., 2023** [99]	ACR II	G1: NXT15906F supplement (herbal formulation pre- pared using *T. indica *seeds and *C. longa *rhizome extracts) (Pharmacological)	30 days (30 days)	*n* = 50 53.1 (9.1); 25.2 (1.4)	Flexion- Instrument- NR WOMAC- Pain, Function, Stiffness, Total
		G2: Blend of *C. longa *and *Boswellia serrata *extracts supplement (Pharmacological)		*n* = 50 51.0 (8.1); 25.3 (1.5)	
		G3: Placebo (No treatment)		*n* = 50 53.4 (9.3); 25.6 (1.3)	
**Rahlf et al., 2019** [100]	Clinical and radiographic NR	G1: Kinesiotape (Non-pharmacological)	3 days (3 days)	*n *= 47 64.7 (7.3); NR	Flexion- Double armed goniometer WOMAC- Pain, Function, Stiffness
		G2: Sham tape (Non-pharmacological)		*n *= 47 65.3 (6); NR	
		G3: Control group/No treatment (No treatment)		*n *= 47 65.4 (7.6); NR	
**Saleem et al., 2022**^**a**^ [101]	Radiographic II to IIII	G1: Pilate exercises (Non-pharmacological)	8 weeks (8 weeks)	*n *= 22 57.6 (6.3); 25.8 (4.2)	Flexion- Goniometer WOMAC- total Pain (NPRS)- Pain
		G2: Isometric exercises (Non-pharmacological)		*n *= 22 55.7 (7.3); 26.9 (4.3)	
**Samaan et al., 2022** [102]	ACR II to IIII	G1: High-intensity laser therapy plus exercise therapy (Non-pharmacological)	2 weeks (2 weeks)	*n* = 20 55.4 (6.3); 29.0 (2.2)	Flexion- Electrogoniometer Pain (VAS)- Pain intensity WOMAC- Total
		G2: Low-intensity pulsed ultrasound plus exercise therapy (Non-pharmacological)		*n *= 20 55.2 (4.8); 29.1 (2.4)	
		G3: Control group/ exercise therapy (Non-pharmacological)		*n* = 20 57.0 (6.4); 29.8 (2.1)	
**Sari et al., 2019** [103]	ACR II to III	G1: Intermittent pneumatic compression, ultrasound therapy, TENS, Exercise (Non-pharmacological)	4 weeks (4 weeks)	*n* = 45 50.8 (9.5); 28.9 (5.8)	Flexion- Universal goniometer Pain (VAS)- Pain intensity WOMAC- Pain, Function, Stiffness
		G2: Cold pack, ultrasound therapy, TENS, Exercise (Non-pharmacological)		*n* = 36 52.3 (7); 28.7 (5.7)	
**Shin et al., 2017** [104]	Clinical and radiographic NR	G1: Theracumin intake (Pharmacological)	8 weeks (8 weeks)	*n* = 13 44.8 (5.4); NR	Flexion- Goniometer Pain (VAS)-Pain WOMAC- Pain, Function, Stiffness, Total
		G2: Theracumin intake combined with exercise (Mixed)		*n *= 12 40.6 (7.7); NR	
**Song et al., 2020**^**a**^ [105]	Medical I to IIII	G1: PNF (Non-pharmacological)	12 weeks (12 weeks)	*n* = 13 68.5 (4.3); NR	Flexion- Goniometer WOMAC- Pain
		G2: Control- No treatment (No treatment)		*n *= 16 67.4 (3.4); NR	
**Sterzi et al., 2016** [106]	ACR II to III	G1: Dietary supplement plus exercise (Mixed)	8 weeks (8 weeks)	*n *= 23 71.3 (8.8); 34.8 (6.4)	Flexion- Instrument- NR Pain (VAS)- Pain at rest, motion WOMAC- Total
		G2: Placebo tablet plus exercise (Mixed)		*n* = 27 71 (8); 34.3 (7.5)	
**Suen et al., 2016**^**a**^ [107]	ACR NR	G1: Placebo laser auriculotherapy and magneto auricular therapy (Non-pharmacological)	6 weeks (6 weeks)	*n* = 11 73 (6.31); NR	Flexion- Standard goniometer Pain (NPRS)- Maximum pain in last 3 days
		G2: Laser auriculotherapy and Placebo magneto auricular therapy (Non-pharmacological)		*n* = 10 72.8 (6.68); NR	
		G3: Laser auriculotherapy plus magneto auricular therapy (Non-pharmacological)		*n* = 13 71.62 (7.75); NR	
		G4: Placebo therapy: Laser switched off mode (Non-pharmacological		*n *= 9 73.86 (8.53) NR	
**Tammachote et al., 2016** [108]	ACR I to IV	G1: Hyaluronic acid injection (Pharmacological)	1 session (2 weeks)	*n* = 50 62.6 (NR); 26.3 (NR)	Flexion- Goniometer Pain (VAS)- Pain
		G2: Triaminolone acetonide injection (Pharmacological)		*n* = 49 61 (NR); 25.8 (NR)	
**Tascιoglu & Öner, 2003** [109]	ACR II to III	G1: Sodium hyaluronate intraarticular injection (Pharmacological)	3 weeks (3 weeks)	*n* = 30 57.4 (6.5); 32.7 (4.1)	Flexion- Instrument- NR Pain (VAS)- Pain at rest, weight bearing, walking
		G2: 6-methyl predinsolone acetate intraarticular injection (Pharmacological)		*n* = 30 60.1 (8.7); 33.3 (4.5)	
**Tascioglu et al., 2010** [110]	ACR II to III	G1: Continuous Ultrasound therapy (Non-pharmacological)	2 weeks (2 weeks)	*n* = 27 59.7 (2.6); 30 (4)	Flexion- Goniometer Pain (VAS)- Knee pain severity WOMAC- total
		G2: Pulsed Ultrasound therapy (Non-pharmacological)		*n* = 28 61.6 (3.7); 30.8 (3.8)	
		G3: Placebo Ultrasound therapy (Non-pharmacological)		*n* = 27 60 (2.8); 28.7 (4)	
**Terzi & Altin, 2017** [111]	Radiographic II	G1: Quadriceps strengthening, ultrasound, TENS, hot pack plus short-wave diathermy (Non-pharmacological)	6 weeks (6 weeks)	*n* = 39 59.5 (8.6); 29.5 (5.4)	Flexion- Goniometer Pain (VAS)- Pain intensity
		G2: Quadriceps strengthening+SWD+TENS+hot pack (Non-pharmacological)		*n* = 38 58.6 (7); 29.4 (5.4)	
		G3: Control group: Quadriceps strengthening only (Non-pharmacological)		*n* = 30 58.1 (7.7); 28.3 (6.0)	
**Torri et al., 1994** [112]	WHO NR	G1: Aceclofenac tablets (Pharmacological)	3 months (3 months)	*n *= 103 57.8 (10.1); NR	Pain- Goniometer Pain (VAS)- Pain
		G2: Piroxican tablets (Pharmacological)		*n* = 102, 56.7 (10.7); NR	
**Wang et al., 2011**^**a**^ [113]	Clinical and radiographic NR	G1: Aquatic exercise therapy (Non-pharmacological)	12 weeks (12 weeks)	*n* = 26, 66.7 (5.6); 26.6 (2.5)	Flexion- Standard goniometer KOOS- Pain, Symptoms, ADL, QOL Sports recreation
		G2: Land based exercises (Non-pharmacological)		*n *= 26, 68.3 (6.4); 25.4 (2.4)	
		G3: Control group- No treatment (No treatment)		*n* = 26, 67.9 (5.9); 26.6 (2.1)	
**Wang et al., 2018** [114]	ACR II to IV	G1: Intraarticular hyaluronic acid and corticosteroid injection (Pharmacological)	1 session (1 session)	*n* = 60, 63.6 (6.2); 25.3 (3.2)	Flexion- Goniometer Pain (VAS)- Pain in the knee WOMAC- total
		G2: Intraarticular hyaluronic injection (Pharmacological)		*n* = 60, 62.5 (6.6); 26 (4.2)	
**Yurtkuran et al., 2006), 2006** [115]	ACR II to III	G1: balenotherapy, pool full of spa water (Non-pharmacological)	2 weeks (2 weeks)	*n* = 29, 52.9 (6.8); 30.8 (6.7)	Flexion- Universal goniometer Pain (VAS)- Pain on movement WOMAC- Pain, Function, Stiffness, Total
		G2: balenotherapy,tap water heated (Non-pharmacological)		*n* = 27 55.5 (6.2); 33.8 (3.9)	
**Zaidi et al., 2009** [116]	Clinical II to III	G1: leeching therapy + traditional unani herbal treatment (Pharmacological)	6 weeks (6 weeks)	*n *= 20, 59.9 (2.8);27.5 (31)	Flexion-Goniometer WOMAC- Pain, Function, Stiffness
		G2: Control group: traditional unani herbal treatment (Pharmacological)		*n* = 20, 57.9 (7.5); 27.5 (33)	

^a^studies did not include in meta-analytic models as no enough data to perform relationships

*OA* osteoarthritis, *SD *standard deviation, *BMI *body mass index, *KL *Kellgren and Lawrence system of grading, *ACR *American College of Rheumatology, *n *= sample size, *G *treatment group, *NR* not reported, *VAS *visual analogue scale, *WOMAC *Western Ontario and McMaster Universities Arthritis Index, *KOOS *Knee injury and Osteoarthritis Outcome Score, *NPRS *numeric pain rating scale, *SF-36 *36-Item Short Form Health Survey, *ADL *activity of daily living, *QOL*, quality of life, *UST* ultra sound therapy, *TENS *transcutaneous electrical nerve stimulation, *SWD *short wave diathermy, *PNF *Proprioceptive Neuromuscular Facilitation, *WHO *World Health Organization

### The risk of bias in included studies

**Fig. 2:**
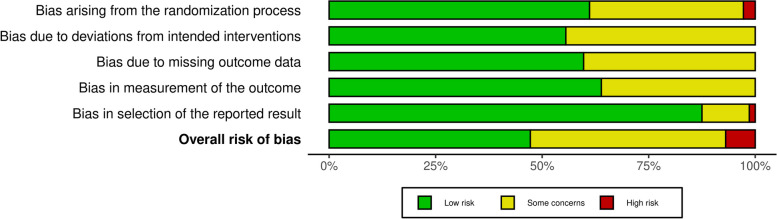
Risk of bias in included studies using RoB 2 tool [35]

The overall risk of bias was low in 47.2% of the studies, while 45.8% had some concerns, and five studies (7%) were of high risk of bias (Fig. 2, Additional file 3). The risk of bias arising from randomisation was low in 61.1% of the studies, while 36.1% had some concerns, and 2.8% of studies were of high risk. The risk of bias due to deviations from intended interventions was low for 55.6%, while 44.4% had some concerns. The risk of bias due to missing outcome data was low in 59.7% of studies, and others (40.3%) had some concerns. The risk bias in measuring the outcomes (63.9%) and selecting the reported results was low (87.5%) in most studies. Therefore, we included all the studies in the data analysis.

### Study characteristics of included studies

All the studies were individually randomised parallel group trials. The mean age ranged from 49 to 71 years. The interventions used in the included studies were exercise, kinesio taping, laser therapy, electrotherapy, shock wave therapy and nonsteroidal anti-inflammatory drugs. The treatment duration ranged from one session to 3 months.

### Changes in knee flexion, pain and function after interventions

The mean *Δ* knee flexion ranged between − 6.4 and 59.8°. *Δ* pain-VAS (/100) at rest, during activity and general ranged from 8.0 to − 39.0, − 7.8 to − 58.8 and 12.8 to − 59.5, respectively. The *Δ* function-WOMAC ranged from 8.8 to − 41.5/100 (Additional file 4A, B).

### Relationship between change in flexion with changes in pain and function

**Fig. 3:**
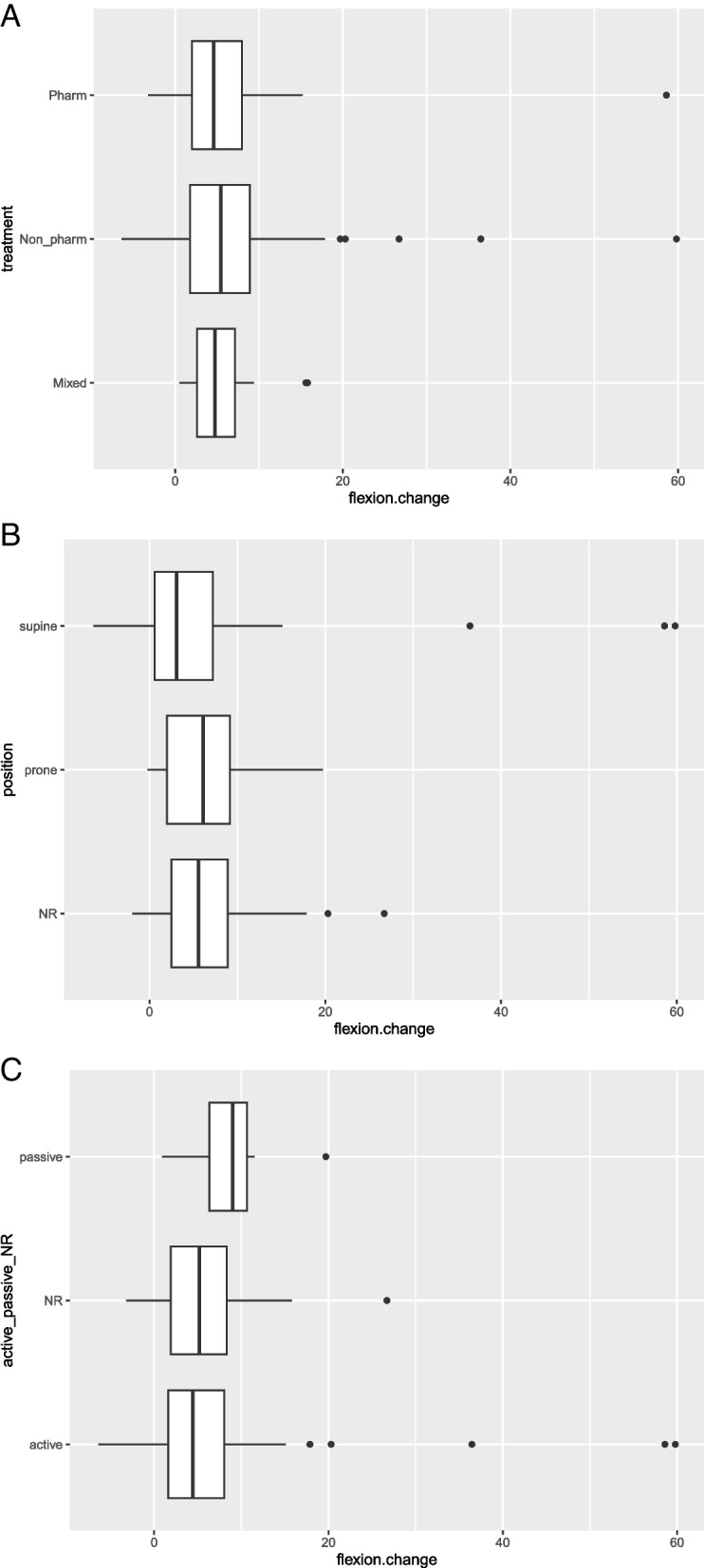
**A** Data distribution of mean change in knee flexion between different nonsurgical interventions. Pharm, pharmacological interventions; Non_pharm, nonpharmacological interventions; mixed, both pharmacological and non-pharmacological interventions; flexion.change, mean change between baseline and immediately after the intervention. **B** Data distribution of mean change in knee flexion between different knee flexion measurement positions. NR, measurement position not reported; prone, prone position; supine, supine position; flexion.change, mean change between baseline and immediately after the intervention. **C** Data distribution of mean change in knee flexion between different methods of flexion assessment (mode). Active, measurement mode-active flexion; NR, measurement mode not reported; passive, measurement mode-passive flexion; flexion.change, mean change between baseline and immediately after the intervention

After examining the sensitivity of knee flexion data to type of interventions, position and mode using data visualisation techniques, we identified there was no apparent effect due to these factors (Fig. 3A, B, C). Therefore, we pooled knee flexion data regardless of the type of intervention, position or mode.

Meta-analyses included 140 intervention arms of 61 studies (*n* = 4516) that reported *Δ* flexion with *Δ* pain-VAS or *Δ* function-WOMAC. Bayesian meta-analytic models found linear relationships between *Δ* flexion with *Δ* pain and *Δ* function (Table 3).

**Table 3:** Parameter estimates for relationships between change in flexion with changes in pain and function

**Model**	**Parameter**	**Posterior mean (°)**	**90% CrI (°)**
Relationship between *Δ* pain at rest with *Δ* flexion	Intercept	− 0.84	− 3.62, 1.95
	Slope	− 0.29	− 0.44, − 0.15
	${\varvec{\tau}}$(study), *n* = 14	2.11	0.92, 3.32
	${\varvec{\tau}}$(intervention arm), *n* = 37	1.90	1.10, 2.87
Relationship between *Δ* pain during activity with *Δ* flexion	Intercept	− 2.08	− 5.78, 1.73
	Slope	− 0.29	− 0.41, − 0.18
	${\varvec{\tau}}$(study), *n* = 10	3.14	0.86, 5.50
	${\varvec{\tau}}$(intervention arm), *n* = 26	2.80	1.65, 4.23
Relationship between *Δ* pain-general with *Δ* flexion	Intercept	− 0.19	− 3.86, 3.34
	Slope	− 0.33	− 0.42, − 0.23
	${\varvec{\tau}}$(study), *n* = 28	8.42	6.56, 10.72
	${\varvec{\tau}}$(intervention arm), *n* = 60	3.79	3.00, 4.77
Relationship between *Δ* function with *Δ* flexion	Intercept	2.99	1.43, 4.48
	Slope	− 0.15	− 0.25, − 0.07
	${\varvec{\tau}}$(study), *n* = 33	2.50	0.81, 3.89
	${\varvec{\tau}}$(intervention arm), *n* = 76	3.65	2.91, 4.51

*CrI*, credible interval; *Δ*mean change between baseline and immediately after the intervention; *Δ*pain is reported in visual analog scale (VAS) where 0 = no pain at all and 100 = worst pain; *Δ*function is measured using the Western Ontario and McMaster Universities Arthritis Index (WOMAC) function subscale, where 0 = best function at all and 100 = worst function. $\uptau$ Heterogeneity estimates using standard deviation (intercept)

Linear relationships between AQ6Δ pain at rest (0-100mm VAS) with Δ flexion was -0.29, (-0.44; -0.15) (β (CrI)). Relationships between *Δ* pain during activity VAS and *Δ* flexion were − 0.29 (− 0.41, − 0.18), and *Δ* general pain-VAS and *Δ* flexion were − 0.33 (− 0.42, − 0.23). The relationship between *Δ* function-WOMAC out of 100) and *Δ* flexion was − 0.15 (− 0.25, − 0.07).

Increased *Δ* flexion was associated with decreased *Δ* pain-VAS and increased *Δ* function-WOMAC. Since the uncertainty of the above relationships was sufficiently low MCIC of knee flexion, using all the above relationships was estimated (Table 4).

**Table 4:** Parameter estimates for MCIC knee flexion

		**MCIC of knee flexion (°)**	
**Model**	Previous (related) MCIC value	Posterior mean (°)	90% CrI (°)
**Relationship between *Δ* pain at rest with *Δ* flexion**	− 19.9 (95% *CI* − 21.6 to − 17.9) (Tubach et al., 2005a)	**5.0**	3.7, 6.4
**Relationship between *Δ* pain during activity with *Δ* flexion**		**3.8**	1.6, 6.1
**Relationship between *Δ* pain-general with *Δ* flexion**		**6.4**	3.6, 9.2
**Relationship between *Δ* function with *Δ* flexion**	− 9.1 (95% *CI* − 10.5 to − 7.5) (Tubach et al., 2005a)	**4.4**	3.3, 5.5
	Function − 17.13 (95% *CI*: − 20.07 to 14.19) (Ornetti, 2011)	**5.6**	4.4, 6.9
	Function − 17.02 (95% *CI* − 20.15 to − 13.9) (Ornetti, 2011)	**5.6**	4.4, 6.9

*MCIC* minimal clinically important change, *CI* confidence interval, *CrI* credible interval, *Δ*mean change between baseline and immediately after the intervention

**Fig. 4:**
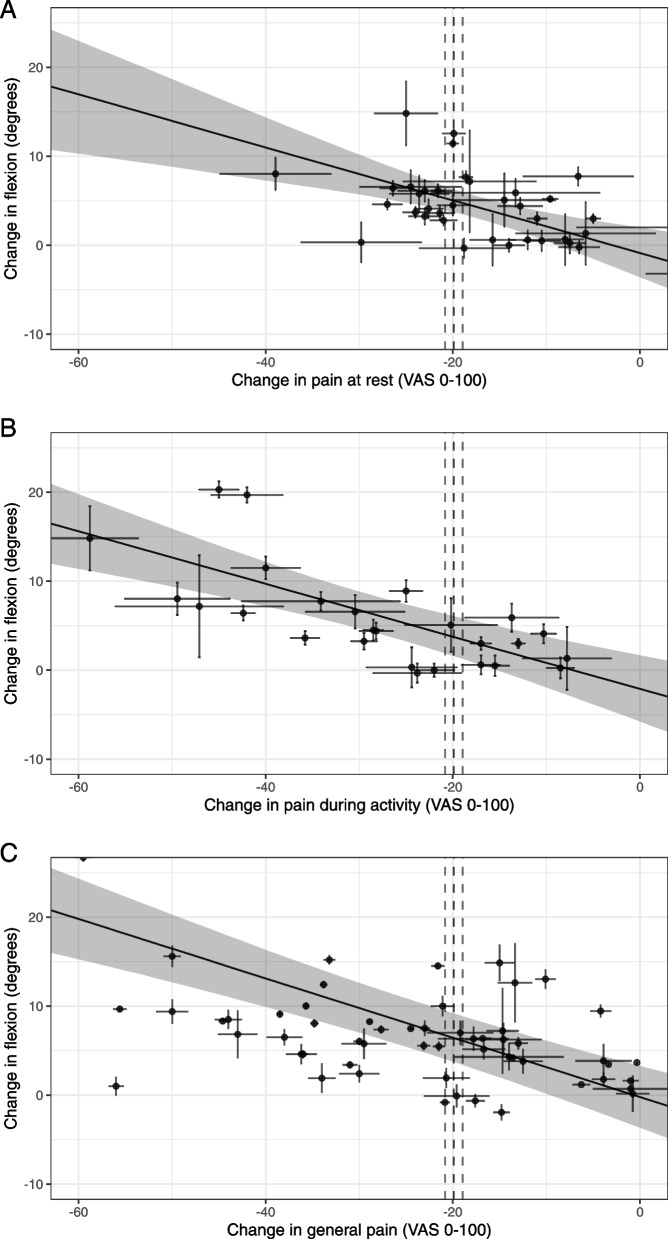
Relationships between changes in **A** pain at rest, **B** during activity, **C** pain-general with change in flexion. *X*-axis represents the change in pain (mean change-pain ± standard error (SE)), and *Y*-axis represents the change in flexion (mean flexion change ± SE). Points represent the observations (estimates from the underlying studies), with horizontal and vertical bars representing the approximate standard error for the predictor and response variable, respectively. The solid line represents the posterior mean of the relationship between the predictor and the response for a hypothetical average study, and the grey field is 90% credible interval (uncertainty), from the meta-regression errors-in-variables model. The vertical dotted lines represent the minimal clinically important change (MCIC) from reference study and its SE (19.9 mm ± 0.94) [45], demonstrating the inference of the corresponding MCIC for flexion

**Fig. 5:**
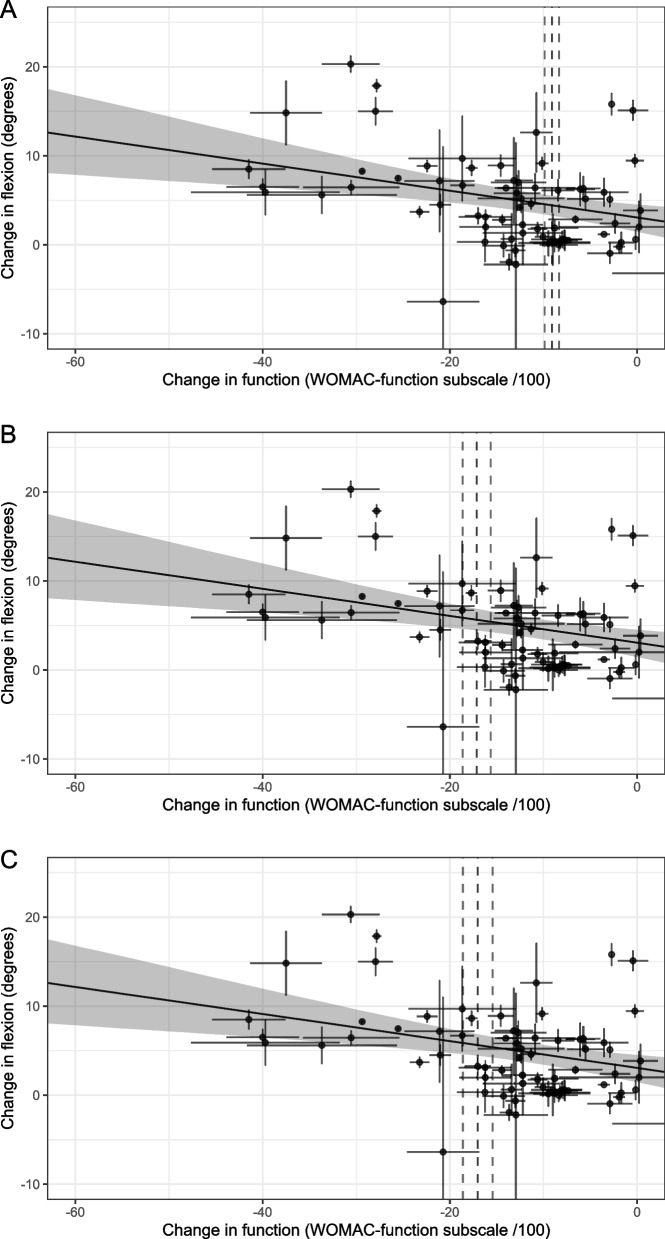
Relationship between change in function with change in flexion. *X*-axis represents the change in function (mean change function ± standard error (SE)), and *Y*-axis represents the change in flexion (mean flexion change ± SE). Points represent the observations (estimates from the underlying studies), with horizontal and vertical bars representing the approximate standard error for the predictor and response variable, respectively. The solid line represents the posterior mean of the relationship between the predictor and the response for a hypothetical average study, and the grey field is 90% credible interval (uncertainty), from the meta-regression errors-in-variables model. The vertical dotted lines represent the minimal clinically important change (MCIC) from reference study _and its SE: **A** − 9.1 + 0.77 [45], **B** − 17.13 + _1.5 [29], **C** − 17.02 + _1.59 [29] demonstrating the inference of the corresponding MCIC for flexion

Point estimates of MCIC of knee flexion ranged from 3.8 to 6.4° with a total range of 90% *CrI* 1.6 to 9.2° (Figs. 4A, B, C and 5A, B, C).

### Relationships for knee flexion with changes in pain and function using knee flexion measured in supine

Due to missing data, a few studies only reported that they measured knee flexion in supine. There were data on supine-active flexion and mode = not reported, and there were no mode = passive data. First, relationships between *Δ* supine-active flexion with *Δ* pain-VAS and *Δ* function-WOMAC were estimated. Second, relationships between *Δ* pooled-supine flexion (position = supine, mode = regardless, active plus not reported) with *Δ* pain-VAS and *Δ* function-WOMAC were estimated (Table 5).

**Table 5:** Parameter estimates for relationships considering supine flexion

Model	Parameter	Posterior mean (°)	90% CrI (°)
**Models using position = supine, mode = active**			
Relationship between *Δ* pain at rest with *Δ* supine-active flexion	Intercept	3.43	− 6.63,12.78
	Slope	− 0.08	− 0.56, 0.37
	${\varvec{\uptau}}$(study) (*n* = 2)	2.22	0.16, 6.12
	${\varvec{\uptau}}$(intervention arms) (*n* = 5)	3.54	1.41, 6.47
Relationship between *Δ* pain during activity with *Δ* supine-active flexion	Intercept	− 3.36	− 12.60, 6.63
	Slope	− 0.27	− 0.56, 0.03
	${\varvec{\uptau}}$(study) (*n* = 2)	1.97	0.13, 5.47
	${\varvec{\uptau}}$(intervention arms (*n* = 5)	2.34	0.27, 5.18
Relationship between *Δ* pain-general with *Δ* supine-active flexion^a^	Intercept	− 4.95	− 18.02, 8.68
	**Slope**	− **0.89**	− **1.09,** − **0.55**
	${\varvec{\uptau}}$(study) (*n* = 4)	25.28	13.88, 42.89
	${\varvec{\uptau}}$(intervention arms) (*n* = 8)	2.59	0.13, 8.63
Relationship between *Δ* function with *Δ* supine-active flexion	Intercept	4.01	1.17, 6.75
	Slope	0.12	− 0.07, 0.32
	${\varvec{\uptau}}$(study) (*n* = 7)	2.49	0.23, 5.76
	${\varvec{\uptau}}$(intervention arms) (*n* = 15)	3.85	2.40, 5.85
**Models using position = supine, mode = pooled**			
Relationship between *Δ* pain at rest with *Δ* pooled-supine flexion	Intercept	− 0.86	− 7.09, 4.54
	Slope	− 0.26	− 0.57, 0.20
	${\varvec{\uptau}}$(study) (*n* = 6)	1.41	0.09, 3.76
	${\varvec{\uptau}}$(intervention arms) (*n* = 16)	2.92	1.19, 4.80
Relationship between *Δ* pain during activity with *Δ* pooled-supine flexion^a^	Intercept	− 3.10	− 7.19, 1.25
	**Slope**	− **0.24**	− **0.37,** − **0.12**
	${\varvec{\uptau}}$(study) (*n* = 5)	1.55	0.14, 3.85
	${\varvec{\uptau}}$(intervention arms) (*n* = 14)	1.88	0.36, 3.62
Relationship between *Δ* pain-general with *Δ* pooled-supine flexion^a^	Intercept	− 0.97	− 11.19, 9.00
	**Slope**	− **0.61**	− **0.93,** − **0.30**
	${\varvec{\uptau}}$(study) (*n* = 7)	15.02	8.12, 23.87
	${\varvec{\uptau}}$(intervention arms) (*n* = 14)	6.54	3.27,11.67
Relationship between *Δ* function with *Δ* pooled-supine flexion	Intercept	4.14	2.35, 5.89
	Slope	0.03	− 0.10, 0.16
	${\varvec{\uptau}}$(study) (*n* = 14)	1.95	0.22, 4.07
	${\varvec{\uptau}}$(intervention arms) (*n* = 33)	3.58	2.52, 4.84

^a^The uncertainty of the relationships was sufficiently low, and they were used to estimate MCIC. *CrI*, credible interval; *Δ*mean change between baseline and immediately after the intervention; *Δ*pain is reported in visual analog scale (VAS) where 0 = no pain at all and 100 = worst pain: *Δ*function is measured using the Western Ontario and McMaster Universities Arthritis Index (WOMAC) function subscale, where 0 = best function at all and 100 = worst function). $\uptau$ Heterogeneity estimates using standard deviation (intercept); ‘position = supine, mode = active’, supine-active flexion data; ‘position = supine, mode = pooled’, pooled-supine flexion data

Considering *Δ* supine-active flexion data, linear relationships between *Δ* supine-active flexion with *Δ* pain-general VAS were − 0.89 (− 1.09, − 0.55) (*β* (*CrI*)). Increased *Δ* supine-active flexion is associated with decreased *Δ* pain-general VAS. The uncertainty of the above estimate was only sufficiently low; MCIC considering supine-active flexion was only estimated based on this relationship (Table 5).

Considering *Δ* pooled-supine flexion data, linear relationships between *Δ* pooled-supine flexion and *Δ* pain-during activity were − 0.24 (− 0.37, − 0.12) (*β* (*CrI*)), and *Δ* pooled-supine flexion and *Δ* pain-general were − 0.61 (− 0.93, − 0.30). *Δ* pooled-supine flexion were associated with decreased *Δ* pain-during activity, and *Δ* pain-general and the uncertainty of these relationships were only sufficiently low. Therefore, MCIC estimates considering pooled-supine flexion were estimated based on these two relationships (Table 6).

**Table 6:** Parameter estimates for MCIC of knee flexion using only reported supine knee flexion

		**MCIC of knee flexion**	
**Model**	**Previous (related) MCIC value**	**Posterior mean (°)**	**90% CrI (°)**
Relationship between *Δ* pain-general with *Δ* supine-active flexion	− 19.9 (*CI* − 21.6 to − 17.9) (Tubach et al., 2005a)	12.8	− 0.0, 26.3
Relationship between *Δ* pain during activity with *Δ* pooled-supine flexion	− 19.9 (*CI* − 21.6 to − 17.9) (Tubach et al., 2005a)	1.7	− 0.5, 4.1
Relationship between *Δ* pain-general with *Δ* pooled-supine flexion	− 19.9 (*CI* − 21.6 to − 17.9a) (Tubach et al., 2005)	11.3	2.4, 20.1

*MCIC* minimal clinically important change, *CI* confidence interval, *CrI* credible interval; *Δ*mean change between baseline and immediately after the intervention

### Estimated MCIC for knee flexion measured in supine

Point estimates of MCIC of knee flexion considering supine-active flexion are 12.8° (− 0.0°, 26.3°) (*β* (*CrI*)).

**Fig. 6:**
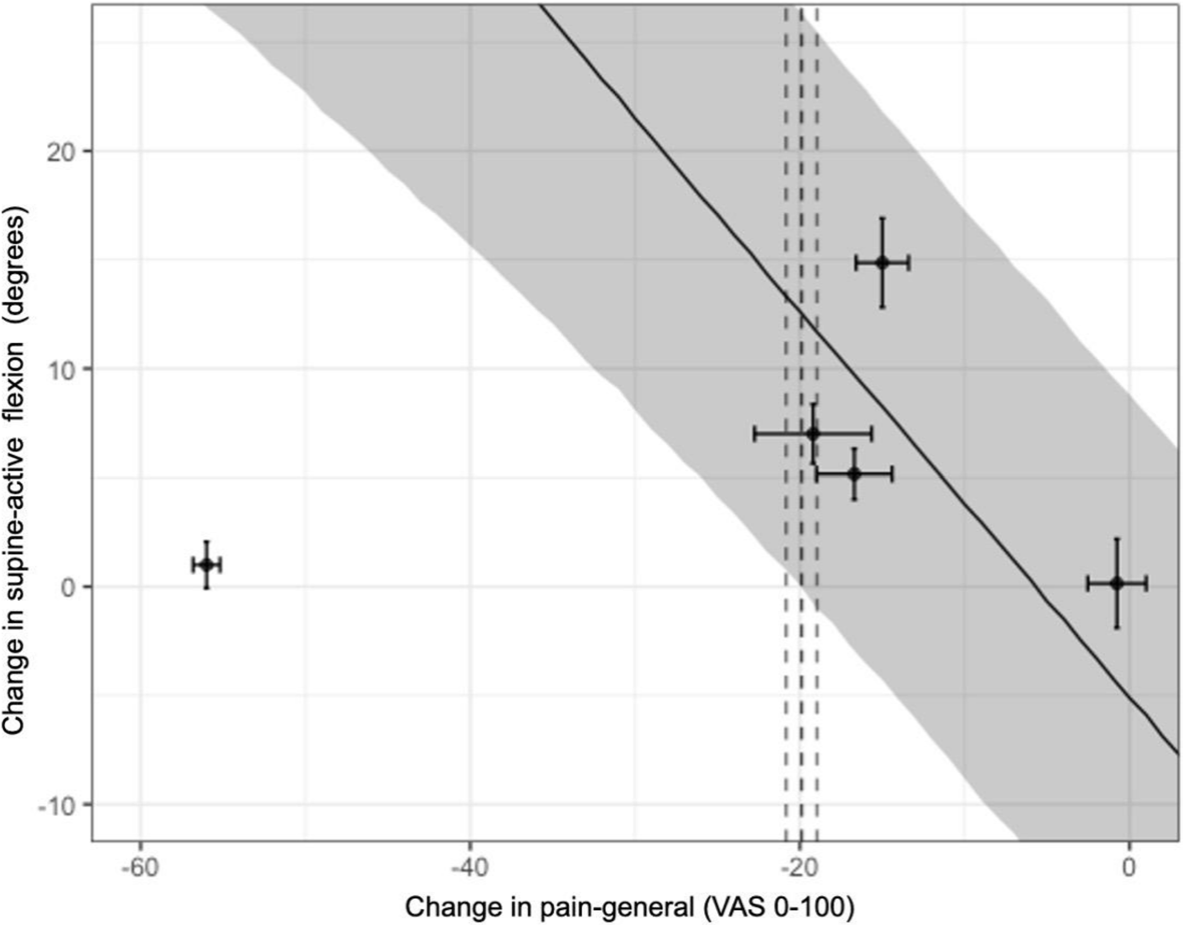
Relationship between change in pain-general with change in supine-active flexion. *X*-axis represents the change in pain (mean change-pain ± standard error (SE)), and *Y*-axis represents the change in flexion (mean flexion change ± SE). Points represent the observations (estimates from the underlying studies), with horizontal and vertical bars representing the approximate standard error for the predictor and response variable, respectively. The solid line represents the posterior mean of the relationship between the predictor and the response for a hypothetical average study, and the grey field is 90% credible interval (uncertainty), from the meta-regression errors-in-variables model. The vertical dotted lines represent the minimal clinically important change (MCIC) from reference study and its SE (19.9 mm ± 0.94) [45], demonstrating the inference of the corresponding MCIC for flexion

**Fig. 7:**
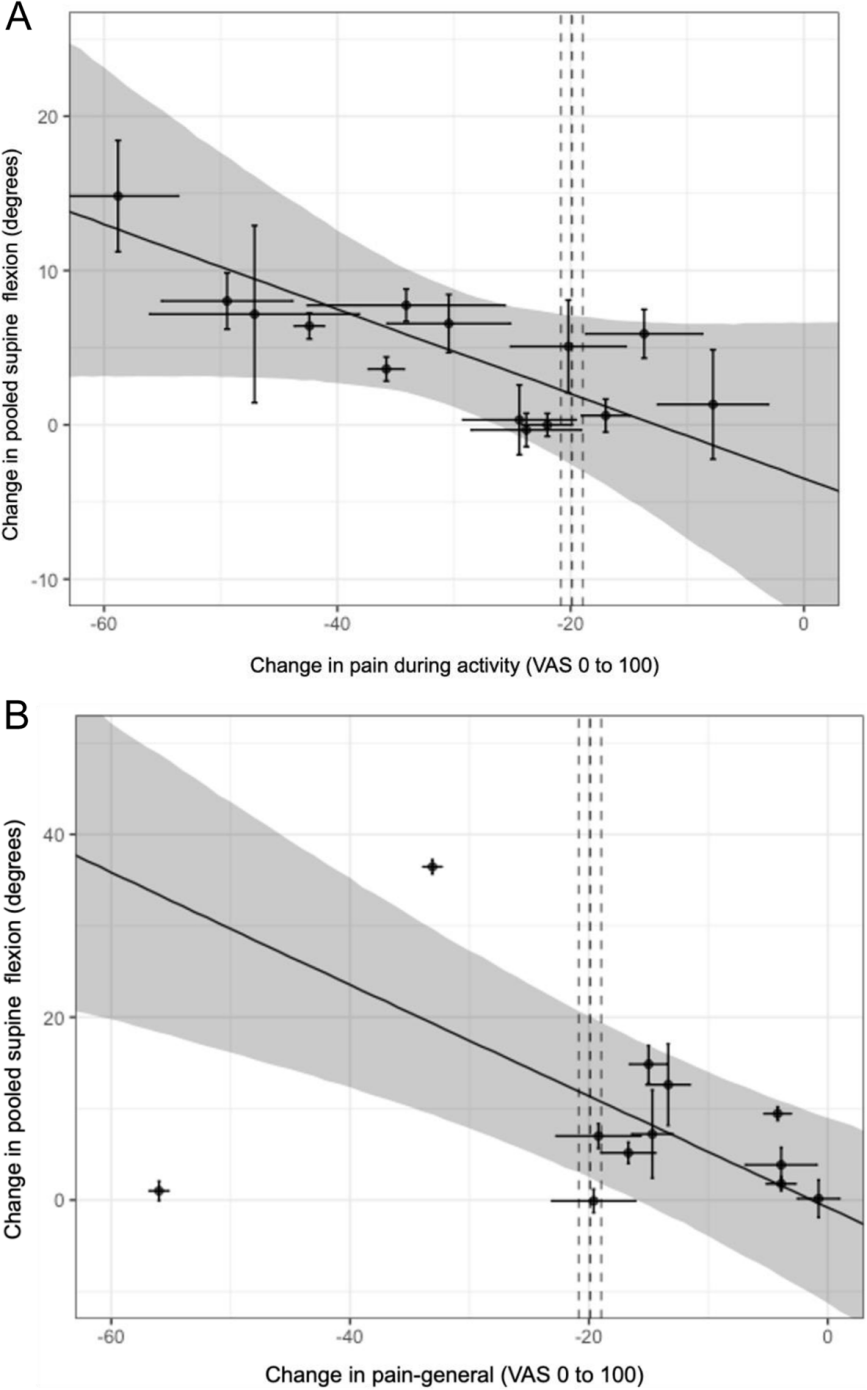
Relationships between changes in pain. **A** During activity. **B** Pain-general with change in pooled-supine flexion. *X*-axis represents the change in pain (mean change-pain ± standard error (SE)), and *Y*-axis represents the change in flexion (mean flexion change ± SE). Points represent the observations (estimates from the underlying studies), with horizontal and vertical bars representing the approximate standard error for the predictor and response variable, respectively. The solid line represents the posterior mean of the relationship between the predictor and the response for a hypothetical average study, and the grey field is 90% credible interval (uncertainty), from the meta-regression errors-in-variables model. The vertical dotted lines represent the minimal clinically important change (MCIC) from reference study and its SE (19.9 mm ± 0.94) [45], demonstrating the inference of the corresponding MCIC for flexion

Point estimates of MCIC of knee flexion considering pooled-supine flexion ranged from 1.7 to 11.3° with a total range of 90% *CrI* − 0.5 to 20.1° (Figs. 6, 7). However, there were still no sufficient studies to draw strong conclusions due to the missing data.

## Discussion

This study provided estimates for MCIC of knee flexion using a meta-analytical approach. We used an innovative method to estimate the MCIC of knee flexion using relationships between *Δ* flexion with *Δ* pain and *Δ* function. We found that the point estimates of knee flexion MCIC ranged from 3.8 to 6.4°. Our MCIC estimates are specific to knee flexion in people with knee OA after non-surgical intervention with an intervention duration of ≤ 3 months. To our knowledge, this is the first study that has implemented relationships between *Δ* flexion with *Δ* pain and *Δ* function and estimated MCIC of knee flexion in people with knee OA. We assumed our methodology was robust, as MCIC was estimated only after the strength of the relationships were assured.

Our estimate for knee flexion MCIC for people with knee OA (3.8 to 6.4°) equates to approximately 3 to 5% of full knee flexion (considering 135° full flexion) [117]. A discussion paper on MCIC stated that MCIC of an outcome measure generally ranged between 6 and 10% of the total score regardless of the outcome measure [118]. Therefore, our estimate is lower than might be expected. However, the estimates are for an angle rather than a survey-based measure, and so the conditions governing these measurements may be different.

While MCIC estimates are specific to the disease condition and outcome measure [4, 119], no previous studies have provided MCIC for knee flexion. One previous study [120] attempted to determine the MCIC of maximum knee flexion during walking using a 3D motion analysis system in people with knee OA following knee arthroplasty; however, they were unable to establish estimates as no association was found between flexion and the anchor questions. This may be because walking requires less than 90° of flexion [117], and so range limitation is less likely to affect walking.

The relationships we found between *Δ* flexion with *Δ* pain and *Δ* function agree with previous studies that reported that flexion improvement was associated with pain relief and functional gain [26]. However, while some studies have found that flexion improvement is related to pain relief [8] and functional improvement [9, 10], others concluded that there is no strong relationship between flexion with pain and function [121]. They suggested that the reason might be that if the patients have already achieved a functional range of motion (more than 110°), flexion improvement may be less important [121].

There are several strengths of this study. Our estimates of MCIC are based on several relationships of *Δ* flexion with *Δ* pain and *Δ* function rather than using just one domain. We combined patient-reported outcome measures (pain and function) with physical outcomes (flexion data), thereby increasing the robustness of our estimates. This accords with recommendations for a holistic approach in disability measures with a combination of physical and patient-reported outcomes [35, 122]. This method for estimating an MCIC may be useful where it is difficult to formulate sensitive anchor questions or where recall bias is an issue [16]. For example, kinematic parameters may be difficult to interpret in an anchor question, and recall bias may be an issue for longer-term outcome measurement. However, any bias in the reference MCIC (MCIC of pain and function in this study) will carry forward into the study estimates (propagation of error) [123], and careful selection of reference estimates should be made.

The results of this study should be interpreted considering its limitations. In contrast to the anchor method, we did not directly acknowledge the patients’ perceptions. Since this is a meta-analytical approach study, these results are based on the results of included studies in this review. Therefore, publication bias and missing data may affect the results. We attempted to estimate MCIC considering only supine flexion data but no adequate studies to draw strong conclusions due to the missing data. We included studies published in English, and grey literature was not searched. Though MCIC can be affected by baseline knee flexion [124], we did not address specific baseline knee flexion, which might have affected the results. Even though we included non-surgical interventions with a treatment duration of ≤ 3 months and addressed the comparability of data before calculating the relationships, clinical variability and confounding caused by between-study factors still may affect the results of this study. Since we used the population level data, this may falsely infer individual data. Furthermore, substantial care has been invested in the internal validity of the analyses leading to MCIC estimates, with respect to data extraction and collation, model and prior specification and model goodness-of-fit evaluation. Much of this information is available to the reader, and we believe that particularly the graphical presentation conveys clearly and transparently the information supporting the results. However, we must note that the external validity of these MCIC estimates is simply unknown. The specific MCIC estimates obtained here have not been externally validated by reference to a repeated study of independent data. Further, we are not aware that meta-analytic modelling of correlated responses has been previously applied for MCIC determination, i.e. the whole approach is novel, so we simply cannot know how well MCIC estimated in this manner would generalise. Though we have carefully considered and evaluated the soundness of the statistical modelling, and from a ground-up perspective the methodology makes sense, replication for similar questions would be necessary to make any judgment about its performance. This is necessarily true for studies using exploratory, rather than established, methods.

Instrument factors should be considered when applying these estimates in clinical and research settings. Flexion range of motion can be measured using a range of goniometers. The most commonly used ‘universal goniometer’ has the minimum detectable change (MDC) ranging from 5 to 10° [125, 126]. However, the digital goniometer is more precise where the MDC is about 2° [127]. If MCIC is less than the MDC of the instrument, the minimal effect cannot be precisely measured using that instrument though the impact is meaningful to the patient [7]. Therefore, it is recommended to use a more precise instrument to measure knee flexion in knee OA studies.

In conclusion, estimated knee flexion MCIC values can be used in clinical and research studies to evaluate treatment efficacy. Though our results are specific to people with knee OA after non-surgical interventions, it is recommended that future studies be undertaken to estimate MCIC as a function of the severity of the knee OA. Our novel meta-analytical approach may be useful for estimating MCIC for other outcome measures where anchor questions are problematic.

### Supplementary Information

**Additional file 1.** Search strategy in MEDLINE (Ebsco) database.

**Additional file 2.** Reference list of included studies.

**Additional file 3.** Risk of bias- included studies.

**Additional file 4.** Changes in knee flexion, pain and function after interventions.

**Additional file 5.** Funnel plots.

## Data Availability

All data generated or analysed during this study are included in this published article and its additional files.

## Abbreviations

OA: Osteoarthritis
ROM: Range of motion
MCIC: Minimal clinically meaningful change
WOMAC: Western Ontario and McMaster Universities Osteoarthritis Index
VAS: Visual analogue scale
PRISMA: Preferred Reporting Items for Systematic reviews and Meta-Analyses
RoB 2: Version 2 of the Cochrane risk-of-bias tool for randomised trials
SE: Standard error
SD: Standard deviation
$$\uptau$$: Heterogeneity variance
CrI: Credible intervals
