# Supplementary material for: Minimal clinically important change of knee flexion in people with knee osteoarthritis after non-surgical interventions using a meta-analytical approach
Source: Syst Rev. 2024 Feb 1;13:50. doi: 10.1186/s13643-023-02393-0 (PMC10832130; doi:10.1186/s13643-023-02393-0)
Supplement: Supplementary file 1 — Additional file 1. Search strategy in MEDLINE (Ebsco) database. [file 13643_2023_2393_MOESM1_ESM.docx]

# **Additional file 1:**

## **Search strategy in MEDLINE (Ebsco) database**

| # | Query |
| --- | --- |
| S1 | knee* OR genu OR tibiofemoral OR patellofemoral |
| S2 | (MM "Knee") OR "knee" OR (MM "Osteoarthritis, Knee") OR (MH "Knee Joint+") |
| S3 | S1 OR S2 |
| S4 | (MH "Osteoarthritis+") OR "osteoarthritis" OR (MM "Osteoarthritis, Knee") |
| S5 | osteoarthr* OR degenerat* |
| S6 | S4 OR S5 |
| S7 | S3 AND S6 |
| S8 | Flexion OR range of motion |
| S9 | Knee injury and Osteoarthritis Outcome Score OR KOOS OR Knee Outcome Score OR KOS OR Numeric* Rating Scale OR NRS OR Intermittent and Constant Osteoarthritis Pain OR ICOAP OR Patient Reported Outcome Measurement Information System OR PROMIS OR Short Form 36 Health Survey OR SF36 OR Visual Analog Scale OR VAS OR Western Ontario and McMaster Universities Arthritis Index OR WOMAC OR Lower Extremity Function* Scale OR LEFS |
| S10 | (MH "Patient-Reported Outcomes+") OR "patient reported outcome measure*" |
| S11 | S9 OR S10 |
| S12 | S7 AND S8 AND S11 |

## **Search strategy in Cochrane database**

ID Search Hits

#1 MeSH descriptor: [Knee] explode all trees

#2 knee* OR genu OR tibiofemoral OR patellofemoral

#3 #1 OR #2

#4 MeSH descriptor: [Osteoarthritis] explode all trees

#5 osteoarthr* OR degenerat*

#6 #4 OR #5

#7 #3 AND #6

#8 MeSH descriptor: [Range of Motion, Articular] explode all trees

#9 Flexion OR range of motion

#10 #8 OR #9

#11 MeSH descriptor: [Patient Reported Outcome Measures] explode all trees

#12 Western Ontario and McMaster Universities Arthritis Index OR WOMAC

#13 Short Form 36 Health Survey OR SF36

#14 Intermittent and Constant Osteoarthritis Pain OR ICOAP

#15 Patient Reported Outcome Measurement Information System OR PROMIS

#16 Numeric* Rating Scale OR NRS

#17 Visual Analog Scale OR VAS

#18 Knee injury and Osteoarthritis Outcome Score OR KOOS

#19 Knee Outcome Score OR KOS

#20 Lower Extremity Function* Scale OR LEFS

#21 patient reported outcome measure*

#22 #11 OR #12 OR #13 OR #14 OR #15 OR #16 OR #17 OR #18 OR #19 OR #20 OR #21

#23 #7 AND #10 AND #22

## **Search strategy in Web of Science database**

**ALL= ((knee* OR genu OR tibiofemoral OR patellofemoral) )**

**ALL=((osteoarthr* OR degenerat*))**

**ALL=(Flexion OR range of motion )**

**ALL=(Knee injury and Osteoarthritis Outcome Score OR KOOS OR Knee Outcome Score OR KOS OR Numeric* Rating Scale OR NRS OR Intermittent and Constant Osteoarthritis Pain OR ICOAP OR Patient Reported Outcome Measurement Information System OR PROMIS OR Short Form 36 Health Survey OR SF36 OR Visual Analog Scale OR VAS OR Western Ontario and McMaster Universities Arthritis Index OR WOMAC OR Lower Extremity Function* Scale OR LEFS )**

All=patient reported outcome measure*

#6= #4 OR #5 308966
**#1 AND #2 AND #3 AND #6**

## **Search strategy in CINAHL database**

| # | Query |
| --- | --- |
| S1 | knee* OR genu OR tibiofemoral OR patellofemoral |
| S2 | (MM "Knee") OR "knee" OR (MM "Osteoarthritis, Knee") OR (MH "Knee Joint+") |
| S3 | S1 OR S2 |
| S4 | (MH "Osteoarthritis+") OR "osteoarthritis" OR (MM "Osteoarthritis, Knee") |
| S5 | osteoarthr* OR degenerat* |
| S6 | S4 OR S5 |
| S7 | S3 AND S6 |
| S8 | Flexion OR range of motion |
| S9 | Knee injury and Osteoarthritis Outcome Score OR KOOS OR Knee Outcome Score OR KOS OR Numeric* Rating Scale OR NRS OR Intermittent and Constant Osteoarthritis Pain OR ICOAP OR Patient Reported Outcome Measurement Information System OR PROMIS OR Short Form 36 Health Survey OR SF36 OR Visual Analog Scale OR VAS OR Western Ontario and McMaster Universities Arthritis Index OR WOMAC OR Lower Extremity Function* Scale OR LEFS |
| S10 | (MH "Patient-Reported Outcomes+") OR "patient reported outcome measure*" |
| S11 | S9 OR S10 |
| S12 | S7 AND S8 AND S11 |
