# Supplementary material for: Minimal clinically important change of knee flexion in people with knee osteoarthritis after non-surgical interventions using a meta-analytical approach
Source: Syst Rev. 2024 Feb 1;13:50. doi: 10.1186/s13643-023-02393-0 (PMC10832130; doi:10.1186/s13643-023-02393-0)
Supplement: Supplementary file 2 — Additional file 2. Reference list of included studies. [file 13643_2023_2393_MOESM2_ESM.docx]

**Additional file 2: Reference list of included studies**

1. Abolhasani M, Halabchi F, Honarpishe R, Cleland JA, Hakakzadeh A: **Effects of kinesiotape on pain, range of motion, and functional status in patients with osteoarthritis: a randomized controlled trial**. *Journal of Exercise Rehabilitation* 2019, **15**(4):603-609.
2. Alfredo PP, Bjordal JM, Dreyer SH, Meneses SRF, Zaguetti G, Ovanessian V, Fukuda TY, Junior WS, Martins RÁBL, Casarotto RA *et al*: **Efficacy of low level laser therapy associated with exercises in knee osteoarthritis: a randomized double-blind study**. *Clinical Rehabilitation* 2012, **26**(6):523-533.
3. Alfredo PP, Junior WS, Casarotto RA: **Efficacy of continuous and pulsed therapeutic ultrasound combined with exercises for knee osteoarthritis: a randomized controlled trial**. *Clinical Rehabilitation* 2020, **34**(4):480-490.
4. Alkhawajah HA, Alshami AM: **The effect of mobilization with movement on pain and function in patients with knee osteoarthritis: a randomized double-blind controlled trial**. *BMC Musculoskeletal Disorders* 2019, **20**(1):452.
5. Alpay K, Sahin M: **Effects of basic body awareness therapy on pain, balance, muscle strength and functionality in knee osteoarthritis: a randomised preliminary trial**. *Disability and rehabilitation* 2022:1‐8.
6. Altınbilek T, Murat S, Yumuşakhuylu Y, İçağasıoğlu A: **Osteopathic manipulative treatment improves function and relieves pain in knee osteoarthritis: A single-blind, randomized-controlled trial**. *Turkish Journal of Physical Medicine and Rehabilitation* 2018, **64**(2):114-120.
7. Arslan Y, Kul A: **Effectiveness Comparison of Extracorporeal Shock Wave Therapy and Conventional Physical Therapy Modalities in Primary Knee Osteoarthritis**. *Turk osteoporoz dergisi* 2022, **28**(2):83‐90.
8. Ashraf A, Riaz S, Arslan HM, Khan RR, Naeem R, Malik A: **Effects of Low Level Laser Therapy on Knee Pain and Functional Status among Patients with Knee Osteoarthritis**. *Pakistan journal of medical and health sciences* 2022, **16**(3):863‐866.
9. Askari A, Ravansalar SA, Naghizadeh MM, Mosavat SH, Khodadoost M, Jazani AM, Hashempur MH: **The efficacy of topical sesame oil in patients with knee osteoarthritis: A randomized double-blinded active-controlled non-inferiority clinical trial**. *Complementary Therapies in Medicine* 2019, **47**:102183.
10. Assar S, Gandomi F, Mozafari M, Sohaili F: **The effect of Total resistance exercise vs. aquatic training on self-reported knee instability, pain, and stiffness in women with knee osteoarthritis: a randomized controlled trial**. *BMC sports science, medicine & rehabilitation* 2020, **12**:27.
11. Aydoğdu O, Sari Z, Yurdalan SU, Polat MG: **Clinical outcomes of kinesio taping applied in patients with knee osteoarthritis: A randomized controlled trial**. *Journal of Back and Musculoskeletal Rehabilitation* 2017, **30**(5):1045-1051.
12. Babaskin DV, Litvinova TM, Babaskina LI: **The Effect of the Phytocomplex Electrophoresis on the Clinical Symptomatology and Quality of Life of Patients with the Knee Joint Osteoarthritis**. *Open access Macedonian journal of medical sciences* 2019, **7**(14):2236-2241.
13. Benedetti MG, Boccia G, Cavazzuti L, Magnani E, Mariani E, Rainoldi A, Casale R: **Localized muscle vibration reverses quadriceps muscle hypotrophy and improves physical function: a clinical and electrophysiological study**. *International Journal of Rehabilitation Research* 2017, **40**(4):339-346.
14. Bhore P, Shinde S: **Effect of multi-component exercises program on pain-related gait adaptations among individuals with osteoarthritis of the knee joint**. *Journal of Education and Health Promotion* 2023, **12**(1).
15. Peréz Busquier M, Calero E, Rodríguez M, Castellon Arce P, Bermudez A, Linares LF, Mesa J, Ffernandez Crisostomos C, Garcia C, Garcia Lopez A *et al*: **Comparison of aceclofenac with piroxicam in the treatment of osteoarthritis**. *Clinical Rheumatology*1997, **16**(2):154-159.
16. Coleman S, Briffa NK, Carroll G, Inderjeeth C, Cook N, McQuade J: **A randomised controlled trial of a self-management education program for osteoarthritis of the knee delivered by health care professionals**. *Arthritis Research & Therapy* 2012, **14**(1):R21.
17. Costa A, Cunha Teixeira V, Pereira M, Mota Ferreira P, Kuplich P, Dohnert M, da Silva Guths J, Boff Daitx R: **Associated Strengthening Exercises to Undenatured Oral Type II Collagen (UC-II). A Randomized Study in Patients Affected by Knee Osteoarthritis**. *Muscles, Ligaments & Tendons Journal (MLTJ)* 2020, **10**(3).
18. Deniz S, Topuz O, Atalay NS, Sarsan A, Yildiz N, Findikoglu G, Karaca O, Ardic F: **Comparison of the Effectiveness of Pulsed and Continuous Diclofenac Phonophoresis in Treatment of Knee Osteoarthritis**. *Journal of Physical Therapy Science* 2009, **21**(4):331-336.
19. Dogan N, Yilmaz H, Ince B, Akcay S: **Is Kinesio Taping Effective for Knee Osteoarthritis? Randomised, Controlled, Double-blind Study**. *Journal of the College of Physicians and Surgeons--Pakistan : JCPSP* 2022, **32**(11):1441‐1447.
20. Donec V, Kubilius R: **The effectiveness of Kinesio Taping(®) for mobility and functioning improvement in knee osteoarthritis: a randomized, double-blind, controlled trial**. *Clinical Rehabilitationl* 2020, **34**(7):877-889.
21. Draper DO, Klyve D, Ortiz R, Best TM: **Effect of low-intensity long-duration ultrasound on the symptomatic relief of knee osteoarthritis: a randomized, placebo-controlled double-blind study**. *Journal of Orthopedic Surgery and Research* 2018, **13**(1):257.
22. Dwyer L, Parkin-Smith GF, Brantingham JW, Korporaal C, Cassa TK, Globe G, Bonnefin D, Tong V: **Manual and manipulative therapy in addition to rehabilitation for osteoarthritis of the knee: assessor-blind randomized pilot trial**. *Journal of Manipulative and Physiological Therapeutic* 2015, **38**(1):1-21.e22.
23. Eftekharsadat B, Jahanjoo F, Toopchizadeh V, Heidari F, Ahmadi R, Ghazani AB: **Extracorporeal Shockwave Therapy and Physiotherapy in Patients With Moderate Knee Osteoarthritis**. *Crescent Journal of Medical and Biological Sciences* 2020, **7**:518-526.
24. Elgendy MH, Elsamahy SA, Mahgoub Mostafa MSE, Hamza MSK: **Efficacy Of Shockwave Therapy Versus Intra-Articular Platelet-Rich Plasma Injection In Management Of Knee Osteoarthritis: A Randomized Controlled Trial**. *International Journal of Pharmaceutical Research (09752366)* 2020, **12**(4):4283-4289.
25. ElGendy MH, Zalabia MM, Moharram AN, Abdelhay MI: **Efficacy of rectus femoris stretching on pain, range of motion and spatiotemporal gait parameters in patients with knee osteoarthritis: a randomised controlled trial**. *BMJ open sport & exercise medicine* 2022, **8**(4):e001459.
26. Fakhari S, Pishghahi A, Pourfathi H, Farzin H, Bilehjani E: **A Comparison Between Low-Level Laser Therapy and Intra-articular Ozone Injection in Knee Osteoarthritis Treatment: A Randomized Clinical Trial**. *Journal of lasers in medical sciences* 2021, **12**(1):e44-e44.
27. Sousa Filho LF, Souza JBd, Almeida GKMd, Jesus ICGd, Oliveira EDd: **Therapeutic ultrasound associated with copaiba oil reduces pain and improves range of motion in patients with knee osteoarthritis**. *Fisioterapia em movimento* 2017, **30**(3):443-451.
28. Fish D, Kretzmann H, Brantingham JW, Globe G, Korporaal C, Moen JR: **A Randomized Clinical Trial to Determine the Effect of Combining a Topical Capsaicin Cream and Knee-Joint Mobilization in the Treatment of Osteoarthritis of the Knee**. *Journal of the American Chiropractic Association* 2008, **45**(6).
29. Forogh B, Mianehsaz E, Shoaee S, Ahadi T, Raissi GR, Sajadi S: **Effect of single injection of platelet-rich plasma in comparison with corticosteroid on knee osteoarthritis: a double-blind randomized clinical trial**. *Journal of sport medicine and physical fitness* 2016, **56**(7-8):901-908.
30. Fung KW, Chow DH, Shae W: **The clinical effects of mobilization with passive ankle dorsiflexion using a passive ankle dorsiflexion apparatus on older patients with knee osteoarthritis: A randomized trial**. *Journal of Back and Musculoskeletal Rehabilitation* 2021, **34**(6):1007-1014.
31. Güngen G, Ardic F, Fιndıkoğlu G, Rota S: **The effect of mud pack therapy on serum YKL-40 and hsCRP levels in patients with knee osteoarthritis**. *Rheumatology international* 2012, **32**(5):1235-1244.
32. Gur A, Cosut A, Sarac AJ, Cevik R, Nas K, Uyar A: **Efficacy of different therapy regimes of low-power laser in painful osteoarthritis of the knee: a double-blind and randomized-controlled trial**. *Lasers in Surgery and Medicine* 2003, **33**(5):330-338.
33. Gurudut P, Jaiswal R: **Comparative Effect of Graded Motor Imagery and Progressive Muscle Relaxation on Mobility and Function in Patients with Knee Osteoarthritis: A Pilot Study**. *Alternative Therapies in Health and Medicine* 2022, **28**(3).
34. Hewlings S, Kalman D, Schneider LV: **A randomized, double-blind, placebo-controlled, prospective clinical trial evaluating water-soluble chicken eggshell membrane for improvement in joint health in adults with knee osteoarthritis**. *Journal of Medicinal Food* 2019, **22**(9):875-884.
35. Ho KK-W, Kwok AW-L, Chau W-W, Xia S-M, Wang Y-L, Cheng JC-Y: **A randomized controlled trial on the effect of focal thermal therapy at acupressure points treating osteoarthritis of the knee**. *Journal of Orthopaedic Surgery and Research* 2021, **16**(1):1-11.
36. Kaya Mutlu E, Mustafaoglu R, Birinci T, Razak Ozdincler A: **Does Kinesio Taping of the Knee Improve Pain and Functionality in Patients with Knee Osteoarthritis?: A Randomized Controlled Clinical Trial**. *American Journal of Physical Medicine and Rehabilitation* 2017, **96**(1).
37. Kaya Mutlu E, Ercin E, Razak Ozdıncler A, Ones N: **A comparison of two manual physical therapy approaches and electrotherapy modalities for patients with knee osteoarthritis: A randomized three arm clinical trial**. *Physiotherapy Theory and Practice* 2018, **34**(8):600-612.
38. Khademi-Kalantari K, Aghdam SM, Baghban AA, Rezayi M, Rahimi A, Naimee S: **Effects of non-surgical joint distraction in the treatment of severe knee osteoarthritis**. *Journal of Bodywork and Movement Therapies* 2014, **18**(4):533-539.
39. Lizis P, Kobza W, Manko G: **Extracorporeal shockwave therapy vs. kinesiotherapy for osteoarthritis of the knee: A pilot randomized controlled trial**. *Journal of Back and Musculoskeletal Rehabilitation* 2017, **30**(5):1121-1128.
40. Kus G, Tarakci E, Ozdincler AR, Ercin E: **Sensory-Motor Training Versus Resistance Training in the Treatment of Knee Osteoarthritis: A Randomized Controlled Trial**. *Clinical Rehabilitation* 2023, **37**(5):636-650.
41. Pawel Lizis PhD D, Grzegorz Manko PhD D, Wojciech Kobza PhD B, Barbara Para PhD B: **Manual therapy with cryotherapy versus kinesiotherapy with cryotherapy for knee osteoarthritis: a randomized controlled trial**. *Alternative Therapies in Health and Medicine* 2019, **25**(4):40-45.
42. Lun V, Marsh A, Bray R, Lindsay D, Wiley P: **Efficacy of Hip Strengthening Exercises Compared With Leg Strengthening Exercises on Knee Pain, Function, and Quality of Life in Patients With Knee Osteoarthritis**. *Clinical Journal of Sport Medicine* 2015, **25**(6).
43. Ma Y-T, Dong Y-L, Wang B, Xie W-P, Huang Q-M, Zheng Y-J: **Dry needling on latent and active myofascial trigger points versus oral diclofenac in patients with knee osteoarthritis: a randomized controlled trial**. *BMC musculoskeletal disorders* 2023, **24**(1):36.
44. Mendes JG, Natour J, Nunes-Tamashiro JC, Toffolo SR, Rosenfeld A, Furtado RNV: **Comparison between intra-articular Botulinum toxin type A, corticosteroid, and saline in knee osteoarthritis: a randomized controlled trial**. *Clinical Rehabilitation* 2019, **33**(6):1015-1026.
45. Moezy A, Kavand S, Angoorani H, Nazari A, Masoudi S: **A randomized controlled trial of two exercise protocols on pain, function, and vastus medialis thickness in women with knee osteoarthritis**. *Journal of Pain Management* 2021, **14**(2):133-144.
46. Mohamed SHP, Alatawi SF: **Effectiveness of Kinesio taping and conventional physical therapy in the management of knee osteoarthritis: a randomized clinical trial**. *Irish journal of medical science* 2022.
47. Nam C-W, Kim K, Lee H-Y: **The influence of exercise on an unstable surface on the physical function and muscle strength of patients with osteoarthritis of the knee**. *Journal of physical therapy science* 2014, **26**(10):1609-1612.
48. Nazari A, Moezy A, Nejati P, Mazaherinezhad A: **Efficacy of high-intensity laser therapy in comparison with conventional physiotherapy and exercise therapy on pain and function of patients with knee osteoarthritis: a randomized controlled trial with 12-week follow up**. *Lasers Med Sci* 2019, **34**(3):505-516.
49. Nidup D, Kietinun S, Niempoog S, Sriyakul K: **Efficacy of rtsa-byugs vs diclofenac gel in relieving knee pain of patients with osteoarthritis of the knee**. *Journal of Health Research* 2020.
50. Oktayoğlu P, Gür A, Yardımeden İ, Çağlayan M, Çevik F, Bozkurt M, Em S, Uçar D, Nas K: **Comparison of the efficacy of phonophoresis and conventional ultrasound therapy in patients with primary knee osteoarthritis**. *Erciyes Medical Journal/Erciyes Tip Dergisi* 2014, **36**(1).
51. Parfitt N, Parfitt D: **The effects of exercise following a corticosteroid injection for knee osteoarthritis: a pilot study**. *Journal of Orthopaedic Medicine* 2006, **28**(2):80-84.
52. Parmigiani L, Furtado RN, Lopes RV, Ribeiro LH, Natour J: **Joint lavage associated with triamcinolone hexacetonide injection in knee osteoarthritis: a randomized double-blind controlled study**. *Clinical Rheumatolgy* 2010, **29**(11):1311-1315.
53. Petrella RJ, Petrella M: **A prospective, randomized, double-blind, placebo controlled study to evaluate the efficacy of intraarticular hyaluronic acid for osteoarthritis of the knee**. *Journal of Rheumatology* 2006, **33**(5):951-956.
54. Pinkaew D, Kiattisin K, Wonglangka K, Awoot P: **Improved WOMAC Score Following Treatment with Nanoparticle Phyllanthus Amarus Phonophoresis Gel for Knee Osteoarthritis**. *Indian Journal of Public Health Research & Development* 2019, **10**(12).
55. Prasad N, Vinay V, Srivastava A: **Efficacy of a proprietary combination of Tamarindus indica seeds and Curcuma longa rhizome extracts in osteoar thritis: a clinical investigation**. *Food & nutrition research* 2023, **67**.
56. Rahlf AL, Braumann K-M, Zech A: **Kinesio taping improves perceptions of pain and function of patients with knee osteoarthritis: a randomized, controlled trial**. *Journal of sport rehabilitation* 2019, **28**(5):481-487.
57. Saleem N, Zahid S, Mahmood T, Ahmed N, Maqsood U, Chaudhary MA: **Effect of Pilates based exercises on symptomatic knee osteoarthritis-A Randomized Controlled Trial**. *Journal of the Pakistan Medical Association* 2022, **72**(01):8-12.
58. Samaan S, Sedhom MG, Grace MO: **A randomized comparative study between high-intensity laser vs low-intensity pulsed ultrasound both combined with exercises for the treatment of knee osteoarthritis**. *International journal of rheumatic diseases* 2022.
59. Sari Z, Aydoğdu O, Demirbüken İ, Yurdalan SU, Polat MG: **A better way to decrease knee swelling in patients with knee osteoarthritis: a single-blind randomised controlled trial**. *Pain Research and Management* 2019, **2019**.
60. Shin Y-A, Suk M-H, Jang H-S, Choi H-J: **Short-term effects of Theracurmin dose and exercise type on pain, walking ability, and muscle function in patients with knee osteoarthritis**. *Journal of exercise rehabilitation* 2017, **13**(6):684.
61. Song Q, Shen P, Mao M, Sun W, Zhang C, Li L: **Proprioceptive neuromuscular facilitation improves pain and descending mechanics among elderly with knee osteoarthritis**. *Scandinavian Journal of Medicine and Science in Sports*, **30**(9):1655-1663.
62. Sterzi S, Giordani L, Morrone M, Lena E, Magrone G, Scarpini C, Milighetti S, Pellicciari L, Bravi M, Panni I: **The efficacy and safety of a combination of glucosamine hydrochloride, chondroitin sulfate and bio-curcumin with exercise in the treatment of knee osteoarthritis: a randomized, double-blind, placebo-controlled study**. *European Journal of Physical and Rehabilitation Medicine* 2016, **52**(3):321-330.
63. .Suen LK, Yeh CH, Yeung SK: **Using auriculotherapy for osteoarthritic knee among elders: a double-blinded randomised feasibility study**. *BMC Journal of International Medical Research 2016,* ***16****(1):1-9.*
64. Tammachote N, Kanitnate S, Yakumpor T, Panichkul P: **Intra-articular, single-shot hylan GF 20 hyaluronic acid injection compared with corticosteroid in knee osteoarthritis: a double-blind, randomized controlled trial**. *JBJS* 2016, **98**(11):885-892.
65. Tascιoglu F, Öner C: **Efficacy of intra-articular sodium hyaluronate in the treatment of knee osteoarthritis**. *Clinical Rheumatology* 2003, **22**(2):112-117.
66. Tascioglu F, Kuzgun S, Armagan O, Ogutler G: **Short-term effectiveness of ultrasound therapy in knee osteoarthritis**. *Journal of International Medical Research* 2010, **38**(4):1233-1242.
67. TERZİ R, ALTIN F: **Evaluation of Short-Wave Diathermy and Ultrasound Treatments as Combined Physical Treatments for Knee Osteoarthritis**. *Journal of Physical Medicine & Rehabilitation Sciences/Fiziksel Tup ve Rehabilitasyon Bilimleri Dergisi* 2017, **20**(3).
68. Torri G, Vignati C, Agrifoglio E, Benvenuti M, Ceciliani L, Raschella B, Letizia G, Martorana U, Tessari L, Thovez G: **Aceclofenac versus piroxicam in the management of osteoarthritis of the knee: a double-blind controlled study**. *Current therapeutic research* 1994, **55**(5):576-583.
69. Wang TJ, Lee SC, Liang SY, Tung HH, Wu SFV, Lin YP: **Comparing the efficacy of aquatic exercises and land‐based exercises for patients with knee osteoarthritis**. *Journal of Clinical Nursing* 2011, **20**(17‐18):2609-2622.
70. Wang SZ, Wu DY, Chang Q, Guo YD, Wang C, Fan WM: **Intra‑articular, single‑shot co‑injection of hyaluronic acid and corticosteroids in knee osteoarthritis: A randomized controlled trial**. *Experimental and Therapeutic Medicine* 2018, **16**(3):1928-1934.
71. Yurtkuran M, Yurtkuran M, Alp A, Nasırcılar A, Bingöl Ü, Altan L, Sarpdere G: **Balneotherapy and tap water therapy in the treatment of knee osteoarthritis**. *Rheumatology international* 2006, **27**(1):19-27.
72. Zaidi S, Jamil S, Sultana A, Zaman F, Fuzail M: **Safety and efficacy of leeching therapy for symptomatic knee osteoarthritis using Indian medicinal leech**. 2009.
